# Switch receptor T3/28 improves long-term persistence and antitumor efficacy of CAR-T cells

**DOI:** 10.1136/jitc-2021-003176

**Published:** 2021-12-01

**Authors:** Songbo Zhao, Chunhua Wang, Ping Lu, Yalin Lou, Huimin Liu, Ting Wang, Shanshan Yang, Ziyou Bao, Lin Han, Xiaohong Liang, Chunhong Ma, Lifen Gao

**Affiliations:** 1 Key Laboratory for Experimental Teratology of Ministry of Education, Shandong Key Laboratory of Infection and Immunity, and Department of Immunology, School of Basic Medical Sciences, Cheeloo College of Medicine, Shandong University, Jinan, Shandong, China; 2 Institute of Marine Science and Technology, Shandong University, Qingdao, Shandong, China; 3 Department of Hematology, Zibo Central Hospital, Zibo, Shandong, China; 4 Biomedical Sciences College & Shandong Medicinal Biotechnology Centre, Shandong First Medical University and Shandong Academy of Medical Sciences, Jinan, Shandong, China

**Keywords:** immunity, immunotherapy

## Abstract

**Background:**

Chimeric antigen receptor (CAR) T cells have been successfully used in tumor immunotherapy due to their strong antitumor responses, especially in hematological malignancies such as B cell acute lymphoid leukemia. However, on-target off-tumor toxicity and poor persistence severely limit the clinical application of CAR-T cell therapy.

**Methods:**

T-cell immunoglobulin mucin domain molecule 3 (TIM-3) was used to develop a second-generation 41BB CD19 CAR linked with a T3/28 chimera, in which truncated extracellular TIM-3 was fused with the CD28 transmembrane and cytoplasmic domains. The efficacy of T3/28 CAR-T cells was evaluated in vitro and in vivo.

**Results:**

We demonstrated that the switch receptor T3/28 preserved the T_CM_ phenotype, improved proliferative capacity, and reduced exhaustion of CAR-T cells, resulting in superior in vitro and in vivo antitumor activity in B lymphoma. Importantly, the switch receptor T3/28 substantially prolonged the persistence of CAR-T cells, and the interleukin-21/Stat3 axis probably contributed to the enhanced cytotoxicity of T3/28 CAR-T cells.

**Conclusion:**

Overall, the T3/28 chimera significantly prolonged the persistence of CAR-T cells, and T3/28 CAR-T cells possessed potent antitumor activity in mice, shedding new light on potential improvements in adoptive T cell therapies.

## Background

Chimeric antigen receptor (CAR)-T cell-based therapy has achieved notable success in cancer treatment, especially for hematological malignancies. To some extent, CAR-T cell therapy has revolutionized the treatment landscape for patients with advanced lymphoid malignancies. Although more than 80% overall response rates have been reported in anti-CD19 CAR-T cell therapies,^1–3^ less than half of the patients have demonstrated durable remissions. The loss of CD19 antigen on malignant B cells usually causes a relapse and recurrence accompanied with common on-target off-tumor side effects. Cytokine release syndrome (CRS) occurs in 57%–97% of patients following CAR-T cell infusion and is the most common life-threatening complication.^2 4 5^ Therefore, the considerable limitations of the widely used CAR design strategies, highlights the necessity to develop a more effective method of novel CAR design to meet fast-growing needs.

As one of the major factors in the antitumor response, T cells exhibit specific cytotoxicity against tumor cells after activation. T cell activation depends on three signals, and the costimulatory receptor CD28 provides the most important second signal for T cell activation. CD28 participates in an intracellular signaling pathway, including unique phosphorylation and transcriptional signaling, glucose uptake, glycolysis, and the production of key cytokines, chemokines, and survival signals for long-term expansion and differentiation of T cells. In addition, CD137, inducible T cell co-stimulator, and other factors combine to promote T cell activation. After T cell activation, inhibitory receptors, also called checkpoints, including programmed death 1 (PD-1), T-cell immunoreceptor with Ig and ITIM domains (TIGIT), lymphocyte-activation gene 3 (LAG3), T cell immunoglobulin mucin domain molecule 3 (TIM-3), are upregulated to maintain T cell homeostasis by preventing T cell**-**mediated cytotoxicity and prompting T cell exhaustion. Moreover, the inflammatory tumor microenvironment usually upregulates ligands for these checkpoints on tumor cells to dampen T-cell activation signaling, which might lead to anergy or exhaustion of T cells in the tumor microenvironment.^6^

Recently, the suppressive effects of immune checkpoints have been successfully switched to activation signals in CAR-T or CAR-natural killer (NK) cells. Shin *et al*^7^ reported that the cytotoxic T-lymphocyte-associated protein 4 (CTLA4)-CD28 chimera, consisting of the extracellular and transmembrane domains of CTLA4 and the cytoplasmic domain of CD28, enhanced the activity of tumor-specific T cells. The PD1-CD28 switch receptor exhibited efficacy against solid tumors by boosting T cell vitality.^8–10^ Hoogi *et al*^11^ designed a TIGIT-CD28 switch receptor that increased cytokine production and T cell activation, as well as substantially enhanced T cell function, which contributed greatly to the improvement of engineered T cell-based immunotherapy. TIM-3, a negative regulator of antitumor immunity, is expressed on activated Th1 cells,^6^ CD8^+^ T cells,^12^ macrophages,^13^ dendritic cells,^14^ and NK cells.^15^ Several ligands of TIM-3, including galectin-9 (Gal-9),^16^ HMGB1,^14^ carcinoembryonic antigen cell adhesion molecule 1 (CEACAM1),^17^ and phosphatidylserine (PtdSer)^18^ have been identified. Binding of TIM-3 to one of its ligands, Gal-9, results in Th1 cell death, suggesting the critical role of TIM-3 in negatively regulating Th1 responses.^19^ TIM-3 also has inhibitory effects on Tc cells, NK cells, and macrophages.^12 15 20^ Compared with PD-1, CTLA4, and TIGIT, ligands of TIM-3 are more widely expressed,^13 16–18^ being expressed on the surface of almost all types of tumors. However, there have been no reports on chimeric switch receptors with TIM-3 in CAR-T cells.

In this study, we generated a lentiviral vector expressing the chimeric switch receptor TIM-3/CD28 (T3/28) linked to a second CAR. In the novel T3/28 chimera, a truncated extracellular TIM-3 was fused with the CD28 transmembrane and cytoplasmic domains to deliver a positive signal instead of the negative signal of TIM-3. The switch receptor T3/28 enhanced CD19 CAR-T cytotoxicity and cytokine production in vitro, and adoptive transfer of T3/28 CAR-T cells to tumor-bearing mice potentiated the therapeutic efficacy of CD19 CAR-T cells. The T3/28 chimeras significantly prolonged the persistence of CAR-T cells. Moreover, the effect of switch receptor T3/28 was further verified in another CAR targeting CD138. Thus, T cell modification with CAR in combination with the T3/28 chimera provides a novel strategy to facilitate adoptive T cell therapy by breaking TIM-3 mediated T cell tolerance.

## Materials and methods

### Cell lines and cell culture

The following cell lines were cultured in RPMI1640 (Invitrogen, Carlsbad, California, USA): Raji, Daudi, Namalwa, RPMI8226, OPM2, and K562 cells, which were purchased from the American Type Culture Collection (ATCC) (Manassas, Virginia, USA). HEK293T cells obtained from ATCC were cultured in Dulbecco’s Modified Eagle’s Medium (DMEM; Invitrogen). DMEM and RPMI1640 media were supplemented with 10% fetal bovine serum (Biowest), 2 mM L-glutamine, 100 U mL^–1^ penicillin and 100 µg mL^–1^ streptomycin. All cells were cultured at 37°C in a humidified 5% CO_2_-containing atmosphere. All cell lines were mycoplasma-free, and validated using flow cytometry for surface markers and functional readouts as needed.

### Vector construction

CARs specific for CD19 were synthesized by Synbio Technologies, as described below. Briefly, the cassette encoding the single-chain antibody targeting CD19, IgG4 Fc spacer, CD8 transmembrane domain, 41BB endodomain, and CD3ζ-chain of the T cell receptor complex were cloned into the pCDH-CMV-MCS-EF1-CopGFP to generate the 19BBz lentiviral vector named 19BBz CAR. We then generated a second lentiviral vector named T3/28 CAR encoding the same 19BBz CAR in combination with a chimeric switch receptor, which was produced by fusing a truncated extracellular TIM-3 with the transmembrane and cytoplasmic domains of CD28 using T2A sequence peptides as described in online supplemental figure S1A. Similarly, a truncated version of T3/28t chimera was synthesized fusing a truncated extracellular TIM-3 with the transmembrane domains of CD28, without cytoplasmic domain.

10.1136/jitc-2021-003176.supp1 Supplementary data

**Figure 1:**
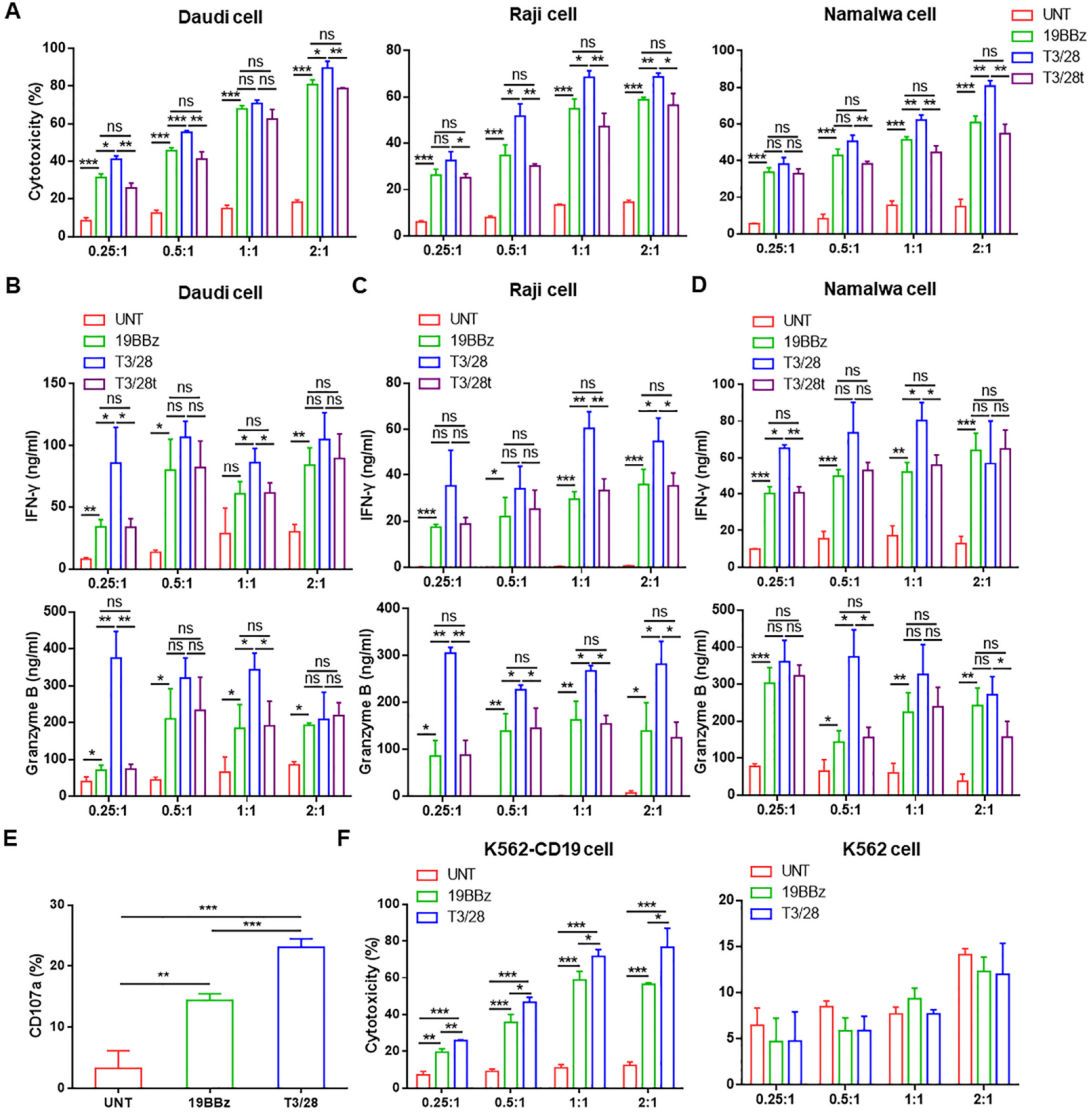
The cytotoxicity and cytokine secretion of T3/28 CAR-T cell are enhanced. (A) The cytotoxicity of transfected CAR-T or control cells to B lymphoma cell lines including Daudi, Raji, and Namalwa was evaluated. Effector cells and target cells (1×10^5^) were co-cultured for 18 hours at E:T ratios of 0.25:1, 0.5:1, 1:1 and 2:1. Then we analyzed the cytotoxicity of UNT, 19BBz and T3/28 CAR-T cells by LDH release assay. IFN-γ and granzyme B released by T cells co-cultured with Daudi (B), Raji (C) and Namalwa (D) were detected using ELISA kit. (E) The CD107a expression of UNT, 19BBz and T3/28 CAR-T cells co-cultured with Daudi cells was analyzed using flow cytometry. (F) The K562 cells and K562 cells overexpressing CD19 (K562-CD19) were co-cultured with UNT, 19BBz, or T3/28 CAR-T cells for 18 hours followed by LDH release assay. Data presented are the mean±SD of three separate experiments. ns means no significant difference, *p＜0.05, **p＜0.01, ***p＜0.001 compared with indicated group. CAR, chimeric antigen receptor; E:T, effector-to-target; IFN, interferon; LDH, lactate dehydrogenase; UNT, untreated T cells.

### Lentivirus production and T cell transduction

The packaging cells of HEK293T were used to produce lentivirus, and the packaging plasmids were **Δ**R, Rev, and VSV-G, which were presented to us by Yun Zhao (The Cyrus Tang Hematology Center, Soochow University). Replication-defective lentiviral particles pseudotyped with VSV-G envelope were produced by transient transfection of HEK293T cells with 10 μg of the gene transfer constructs, 6.5 μg **Δ**R, 3.5 μg VSV-G, and 2.5 μg Rev, using Lentifit (Hanbio, China) transfection reagents. After 12 hours, the supernatants were replaced with fresh culture medium. Viral supernatants were harvested at 48 and 72 hours. After filtering through 0.22 µm filters to increase the concentration, supernatants were either used immediately for transduction or were aliquoted and stored at **−**80°C until use.

To generate CAR-T cells, mononuclear cells from peripheral blood derived from normal human donors were collected and cultivated in TexMACS GMP medium supplemented with 50 IU mL^−1^ IL-2 at a density of 1×10^6^ cells mL^−1^, and activated with anti-CD3/CD28 beads (Miltenyi Biotec, Bergisch Gladbach, Germany). After 48 hours of activation, T cells were transduced with viral supernatants (multiplicity of infection (MOI)=20). Post-expansion, CAR-T cells were harvested and green fluorescent protein (GFP) expression was determined using flow cytometry (Cytoflex, Beckman Coulter).

### T cell isolation and ex vivo T cell proliferation assay

All studies were conducted in accordance with the Chinese Common Rule Ethical Guidelines. Peripheral blood samples were obtained from several healthy donors. The peripheral blood mononuclear cells (PBMCs) were isolated by density gradient centrifugation using Ficoll-Paque (GE Healthcare) and T cells were cultured in TexMACS GMP medium (Miltenyi Biotec). T cells were stimulated with CD3/CD28 activation and proliferation beads (Miltenyi Biotec) at a 1:1 ratio (T cell: bead) for 48 hours in TexMACS GMP medium supplemented with 10% fetal bovine serum, 2 mM L-glutamine and 50 IU mL^−1^ IL-2.

Forty-eight hours after transfection, 5×10^4^ CAR-T or T cells were plated and co-cultured with Namalwa cells at an effector-to-target (E:T) ratio of 5:1 in a total volume of 1 mL. Three days later, all cells in the well were collected, counted, resuspended in fresh medium, and added to a new plate seeded with 1**×**10^4^ tumor cells. The number of T cells was recorded every 3 days. For the CCK8 assay, normalized numbers (2×10^4^) of CAR-T or untreated T cells (UNT) were co-cultured with 10 pg mL^–1^ interleukin (IL)-21 small interferring RNA (siRNA) per well in complete TexMACS GMP medium in 96-well plates in triplicate.

### Flow cytometry and western blotting

Mononuclear cells were harvested from the spleen, liver, kidney and peripheral blood were obtained with the visceral organs processing grinding or erythrocyte-lysing. Tumor cells or lymphocytes were washed and analyzed in ice cold phosphatic buffer solution (PBS). All antibodies were titrated for optimal staining. CAR expression was measured using GFP or Alexa-Fluor-647-conjugated goat anti-mouse Fab (Jackson ImmunoResearch). The phenotype of primary cells and cell lines was determined using the following anti-human antibodies: CD3-APC (clone HIT3a, BioLegend), CD3-Pcy5.5 (clone OKT3, BioLegend), CD4-PC7 (clone OKT4, BioLegend), CD8-BV421 (RPA-T8, BioLegend), CD19-FITC (clone HIB19, BD), CD19-APC (clone HIB19, BD), CD25-APC (clone 2A3, BD), CD107a-APC (clone H4A3, BioLegend), TIM3-APC (clone F38-2E2, BioLegend), LAG3-APC (clone 7H2C65, BioLegend), PD1-PE/dazzle (clone EH12.2H7, BioLegend), PD1-PC5.5 (clone EH12.1, BD), Foxp3-PE (clone 259D, BioLegend), Tigit-PC5.5 (clone A15153G, BioLegend), CD127-PE (clone HIL-7R-M21, BD), CD27-PC5.5 (clone M-T271, BD), CD28-PE (clone CD28.2, BD), CD45RO Alexa Fluor 700 (clone UCHL1, BD), CD62L-PE (clone DREG-56, BD), CD66 (Ceacam1)-BV421 (clone ASL-32, BioLegend), Gal-9-PE (clone ASL-32, BioLegend), Ki67-BV421 (clone Ki-67, BioLegend), and CD69-APCcy7 (clone PN50, BioLegend). After staining, all cells were incubated at room temperature (RT) for 20–25 min, washed three times with PBS, and analyzed on a flow cytometer (Cytoflex, Beckman Coulter). For intracellular staining, cells were fixed and permeabilized using the Intracellular Fixation and Permeabilization Buffer set (Invitrogen) according to the manufacturer’s protocol. All samples were analyzed using FlowJo software (V.10.1, TreeStar) and GraphPad Prism Software V.6.01.

Whole-cell lysates of CAR-T cells were generated by lysing 5×10^6^ washed cells in 200 µL of RIPA buffer, separated using SDS-PAGE, and transferred onto PVDF membranes. Protein concentrations were determined using the BCA assay (Thermo Fisher Scientific). The following primary antibodies were used: anti-CD3ζ (BD), anti-BCL-xL (Proteintech), anti-BCL-2 (Proteintech), anti-caspase-3 (ABclonal), anti-pStat3-S727 (ABclonal), anti-Stat3 (Proteintech), anti-pStat5-Y694 (ABclonal), anti-Stat5A/B (ABclonal), anti-GAPDH (Proteintech), and anti-actin (Proteintech). After blocking, the blots were incubated overnight at 4°C with the corresponding primary antibodies at dilutions recommended by the suppliers, followed by incubation with HRP-conjugated secondary antibodies (Solarbio) at RT for 1 hour. Images were captured using a visualizer (Tanon 4600, TannonBio, Shanghai, China). Image processing was performed using Image J software. Actin was used as loading control.

### qPCR and primers

Quantitative PCR (qPCR) was used to quantify the expression levels of certain candidate genes. Total messenger RNA (mRNA) was extracted from cells using the TRIzol reagent (TIANGEN, Beijing, China) and reverse transcribed into complementary DNA (cDNA) using the RevertAid First Strand cDNA Synthesis Kit (Thermo Fisher Scientific), according to the manufacturer**’**s instructions. All reactions were performed with TaqMan Fast Universal PCR Master Mix (Vazyme) on a real-time PCR machine (CFX Connect), using the following primer pairs with noted amplification factors (mean±SE.e.m.): CD19 CAR (Forward: 5’-GACTACAGCCTGACCATCTCCAA; Reverse: 5’-GCTTTCTTGCAGCTTCACCTCGC-3’); human T-bet (Forward: 5’-TGTTGTGGTCCAAGTTTAATCAG-3’; Reverse: 5’-CCACAGTA AATGACAGGAATGG-3’); human Blimp-1 (Forward: 5’-CAGCTCGCCCACCTGCAGAA-3’; Reverse: 5’-GCCGCAGCGCAGTTCCCTTT-3’); human LEF1 (Forward: 5’-CGACGCCA AAGGAACACTGAGATC-3’; Reverse: 5’-GCACGCAGATATGGGGGGAGAAA-3’); human TCF7 (Forward: 5’-CTGGCTTCTACTCCCTGACCT-3’; Reverse: 5’-ACCAGAACCTAGCAT CAAGGA-3’); Human IL-21 (Forward: 5’-TAGAGACAAACTGTGAGTGGTCA-3’; Reverse: 5’-GGGCATGTTAGTCTGTGTTTCTG-3’); Human TOX (Forward: 5’-TATGAGCATGACA GAGCCGAG-3’; Reverse: 5’-GGAAGGAGGAGTAATTGGTGGA-3’); Human TOX2 (Forward: 5’-AGAGCGAGAACAACGAAGACT-3’; Reverse: 5’-TGGCCTGATAGGAGTAGGCAG-3’); Human TOX3 (Forward: 5’-CCTGCCAGCCTGGACTTC-3’; Reverse: 5’-GAGGAGGCGTGAT TGGTGG-3’); Human TOX4 (Forward: 5’-TGACAATTACCTGACGATCACAG-3’; Reverse: 5’-TCCAAGGAGATAGGTGGGATTTC-3’); Human CEACAM1 (Forward: 5’-acaactcca accctgtggaggac-3’; Reverse: 5’-TTCACACTCATAGGGTCCTGTGT-3’); Human Gal-9 (Forward: 5’-gtttgctgtgaactttcagact-3’; Reverse: 5’-CTTGAAATCTGAGCTCT GCACCA-3’); Human HMGB1 (Forward: 5’-ttatgaaagagaaatgaaaacct-3’; Reverse: 5’-GCAGCAATATCCTTTTCGTATTT-3’); Human β-actin (Forward: 5’-GGCATCGTGAT GGACTCCG-3’; Reverse: GCTGGAAGGTGGACAGCGA-3’). Delta CT calculations were relative to β-actin and corrected for PCR efficiency. All the primers used were commercially available.

### In vitro killing experiment

In the in vitro killing experiment, the CytoTox 96 Non-Radioactive Cytotoxicity Assay (Promega, USA) was used to assess the killing of target cells by measuring lactate dehydrogenase (LDH) release from tumor cells according to the manufacturer’s instructions. Target tumor cells (1×10^5^) were prepared in cell culture medium and co-incubated with CAR-T cells for 18 hours at E:T ratios of 0.25:1, 0.5:1, 1:1, and 2:1 in a total volume of 500 µL. In cases of unequal transduction efficiency, untreated T cells were supplemented to ensure that both the number of CAR-T cells and the total number of T cells remained consistent across the CAR-T cell groups. After 18 hours, 50 µL of killer cell supernatants from each group were placed in 96-well plates, and 50 µL of CytoTox 96 reagent was added to each well. The plate was covered with foil to protect it from light and incubated for 30 min at RT. Then 50 µL of the stop solution was added to each well. Absorbance was recorded at 490 nm for each group with a microplate reader (TECAN, infinit M200pro) after 1 hour of reaction. The spontaneous release of effector and target cells and maximum release of target cells were also measured. The killing rates for each group were calculated using the following formula:

Killing rate (%) = (experimental group cell death − apoptotic effector cell death − apoptotic target cell death)/(total target cell death − apoptotic target cell death)×100%

In an alternate method of measuring cytotoxicity of CAR-T cells to targets, 1×10^5^ target cells and CAR-T cells were co-incubated with different amounts of recombinant human TIM-3 Fc chimera (R&D systems) with 0, 1, and 5 µg in a total volume of 1 mL for 1 hour, respectively. The tumor cells were then co-cultured with CAR-T cells for 8 hours at an E:T ratio of 1:1 in a total volume of 500 µL. Finally, supernatants were harvested and analyzed using the CytoTox 96 reagent.

### Degranulation assay and cytokines assay

T cells (2×10^5^ cells) were co-cultured with target cells at a ratio of 5:1 for 6 hours at 37°C and 5% CO2 with GolgiStop (BD) and APC-conjugated anti-CD107a antibody in 48-well round-bottom plates. Then, the positive rates of T cells were analyzed using a flow cytometer (Cytoflex, Beckman Coulter).

T cells were co-cultured with tumor cells (1×10^5^) in 24-well plates at E:T ratios of 0.25:1, 0.5:1, 1:1, and 2:1 in a total volume of 500 µL without the addition of exogenous cytokines. After incubation for 18 hours, supernatants were collected and used for ELISA. In addition, serum obtained from mice on day 17 was used for the ELISA. The human interferon (IFN)**-**γ, granzyme B, and IL-21, were measured using specific ELISA kits (Multi Sciences) following the manufacturer’s instructions. The human soluble Gal-9 in supernatants obtained from Daudi or Raji cells was analyzed using specific ELISA kits (CUSABIO). The results represent the mean±SD of three separate experiments.

The other cytokines, including IL-1β, IL-2, IL-4, IL-6, IL-8, IL-10, IL-12, IL-17, MCP-1, MIP-1α, GM-CSF, TNF-α, perforin, and TGF-β, were detected using microfluidic immunofluorescence technology. Briefly, a polydimethylsiloxane (PDMS) template was overlaid onto graphene oxide quantum dot (GOQD)-coated glass substrate and high-density antibody barcode microarray was generated by flowing capture antibodies along parallel microchannels on the PDMS chip for 3 hours. GOQD glass substrate was then detached from the PDMS chip and blocked with 3% bovine serum albumin (BSA) for 10 min. Then, the antibody barcode microarray substrate was aligned with the sample loading PDMS chip with multiple detection units for sample loading. Subsequently, the collected serum or supernatants were added into individual detection units, followed by incubation for 20 min. After incubation, sample residues were removed from each detection unit and the sample loading PDMS layer was peeled off from the antibody microarray substrate in 1% BSA, and the substrate was flushed with 1% BSA. Further, 300 µL fluorescence-conjugated detection antibody complex at a concentration of 10 μg mL^−1^ was loaded onto the substrate and dispersed over the entire substrate area. After incubation for 20 min, the substrate was washed with 1% BSA, DPBS, and DI water, and then blown dry. A Genepix 4400A scanner was used to obtain scanned fluorescence images in fluorescein isothiocyanate APC (635 nm) channel. The image was analyzed using GenePix Pro software by aligning signals with an array template. The fluorescence intensities were then extracted and exported into Excel files.

### H&E staining

The visceral organs were obtained from the euthanized mice and immediately fixed in formalin overnight. Fixed tissues were dehydrated, embedded in paraffin, sectioned, and dewaxed. Then, dewaxed slides were directly subjected to H&E staining and the tumors were observed under an upright fluorescence microscope.

### Clinical material

For clinical specimens, fresh tumor samples from patients with de-identified B lymphoma were obtained from Zibo Central Hospital (Zibo, China). PBMCs were isolated by density gradient centrifugation over Ficoll-Paque (GE Healthcare), and B cell subsets were detected using anti-CD19 antibody. The samples were used, and the percentage of CD19-positive B cells was greater than 80%.

### RNA sequencing analysis

Seven days after stimulation with CD3/CD28 activation and proliferation beads (Miltenyi Biotec), the UNT, 19BBz, and T3/28 CAR-T cells were harvested. RNA was isolated from the above populations using the RNeasy Mini Kit (Qiagen). Paired-end libraries were synthesized using the TruSeq RNA Sample Preparation Kit (Illumina, USA) following the TruSeq RNA Sample Preparation Guide. Library construction and sequencing were performed by Sinotech Genomics (Shanghai, China). Briefly, poly A-containing mRNA molecules were purified using poly T oligo-attached magnetic beads. Paired-end sequence files (fastq) were mapped to the reference genome using Hisat2 (Hierarchical Indexing for Spliced Alignment of Transcripts, V.2.0.5). The output SAM files were converted to binary alignment/map files and sorted using SAM tools (V.1.3.1).

### Animal experiments

All mouse studies were conducted in accordance with protocols approved by the Institutional Animal Care and Use Committee of Shandong University. The experiments were conducted at the Animal Experiment Center of Shandong University under specific pathogen-free conditions. Female mice 5–6 week-old, B-NDG (NOD-Prkdc^scid^ Il2rg^tm1^), purchased from Beijing Biocytogen with an average weight of 16–18 g were used. The mice were intravenously injected with 1×10^6^ Daudi-Fluc cells suspended in 200 µL PBS and randomly allocated to the following four groups, with five to six mice in each group: (i) injection with 200 µL of PBS during each treatment; (ii) injection with 2×10^6^ UNT cells (200 µL) during each treatment; (iii) injection with 2×10^6^ 19 BBz CAR-T cells during each treatment (200 µL); and (iv) injection with 2×10^6^ T3/28 CAR-T cells (200 µL) during each treatment. CAR-T cells were administered *via* tail vein injections, and three treatments were provided 4, 8, and 12 days after tumor cell injection. The body weight of each mouse was measured three times per week. The mice were euthanized in a humane manner when death was imminent or when the posterior limb was paralyzed. In addition, the mice in the parallel experiment were euthanized. On day 14 after tumor inoculation (the third day after the third CAR-T cell infusion), blood, spleen, liver, kidney, and bone marrow cells were isolated from mice from each experimental group. Erythrocytes were lysed with red blood cell lysis buffer (0.15 M NH4Cl, 10 mM NaHCO3, and 0.1 mM EDTA), and cells were stained with antibodies specific for CD3, CD25, CD27, LAG3, Tigit and CD19 and analyzed by flow cytometry. The visceral organs were fixed in 4% neutral buffered formaldehyde for several days and processed for paraffin sectioning using standard protocols.

For the RPMI8226-bearing mouse model, the mice were subcutaneously injected with 5×10^6^ RPMI8226 cells into the right shoulder. Treatment was performed when the tumor size reached 20 mm^3^. The mice were randomly allocated to the following four groups, with six mice in each group: (i) injection with 200 µL of PBS during each treatment; (ii) injection with 5×10^6^ UNT cells (200 µL) during each treatment; (iii) injection with 5×1 0^6^ 138 BBz CAR-T cells during each treatment (200 µL); and (iv) injection of 5×10^6^ 138-T3/28 CAR-T cells (200 µL) during each treatment. CAR-T cells were administered *via* tail vein injections, and two treatments were provided 6 and 9 days after tumor cell injection. The tumor size was calculated using the following formula: 4π/3 × (tumor length/2) × (tumor width/2)^2^.

The in vivo persistence experiment was conducted using 5–6 week-old female B-NDG mice. The mice were intravenously injected with 5×10^5^ T cells (UNT, 19BBz, or T3/28 CAR-T cells) suspended in 200 µL PBS with five to six mice in each group. Mice were intravenously injected with 2×10^4^ Namalwa-Fluc cells on day 6. Eight days after Namalwa-Fluc cell treatment, mice were re-challenged with 2×10^4^ Namalwa-Fluc and the previous step was repeated several times until all mice died. Peripheral blood was obtained every 5 days *via* venous blood collection, erythrocytes were lysed with red blood cell lysis buffer and mononuclear cells were stained with antibodies specific for CD3, CD4 and CD8 and analyzed by flow cytometry. For the other model, five mice in each group were intravenously injected with 5×10^6^ T cells (UNT, 138BBz, or 138-T3/28 CAR-T cells) suspended in 200 µL PBS. Then, mice were intravenously injected with 1×10^5^ RPMI8226 cells on day 3, 6, and 9. Along with detection, CAR-T cells from mononuclear cells in tumors were analyzed using flow cytometry.

### In vivo bioluminescence imaging

We used D-luciferin (BioVision) in PBS (1.5 mg mL**^−^**^1^) as a substrate for F-luc (for imaging Daudi-Fluc or Namalwa-Fluc cells) following the manufacturer’s protocols. Tumor progression was monitored weekly by bioluminescence imaging using an In Vivo Imaging System (IVIS) Spectrum Imaging System (PerkinElmer). Living Image V.4.5.5 (PerkinElmer) was used to acquire (and later quantify) the data 10 min after intraperitoneal injection of D-luciferin into animals that were anesthetized with 150 mg kg**^−^**^1^ of 1% pentobarbital sodium (Sigma-Aldrich). The acquisition time ranged from 1 s to 1 min. Imaging settings were kept the same throughout the duration of the experiment.

### Statistical analysis

The in vitro experiments were repeated at least three times for each group, and Student’s t*-*test was used to compare quantitative data (mean±SD) between samples. Analysis of variance or log-rank (Mantel-Cox) test for mouse survival data were performed using GraphPad Prism V.7 software. Differences were considered significant when p value was <0.05.

## Results

### Design and identification of T3/28 CAR

First, we designed a CAR with a CD19-specific single-chain variable fragment consisting of heavy and light chains, and a signal peptide sequence (MLLLVTSLLLCELPHPAFLLIP) was fused to a second generation CAR backbone containing an IgG4 spacer and cytoplasmic domains of 41BB and CD3zgenes, which was called 19BBz. Subsequently, we generated the T3/28 CAR consisting of traditional CD19 CAR described above and the switch receptor T3/28 or T3/28t (truncated T3/28 CAR) (online supplemental figure S1A). We transduced these recombinants or control vectors into donor-derived T cells and determined transduction efficiency in T cells based on GFP expression 6–8 days post transduction. The GFP expression rates on 19BBz CAR-T cells, T3/28 CAR-T cells and T3/28t CAR-T cells were 50%–90%, 55%–90%, and 50%–90%, respectively (online supplemental figure S1B and C), indicating that the three CARs could be successfully transduced into human primary T cells from donors with a steady transduction efficiency. Western blotting and qPCR were performed to determine whether CAR transduction resulted in an increase in protein and mRNA expression compared with UNT (online supplemental figure S1D and E), and the cell surface levels of TIM-3 were assayed using flow cytometry (online supplemental figure S1F).

### Switch receptor T3/28 enhances T cell cytotoxicity and cytokine secretion in vitro

Some chimeric switch receptors can promote the tumor-lysing ability of T cells.^7–9^ Therefore, we assessed the impact of the switch receptor T3/28 on CD19 CAR-T cell cytotoxicity in three human B lymphoma cell lines (Daudi, Raji, and Namalwa) that highly express CD19. Compared with 19BBz, T3/28t and UNT cells, the T3/28 CAR-T cells demonstrated impressive cytotoxicity against CD19-positive cells in a dose-dependent manner (figure 1A). Surprisingly, the killing rate of T3/28 CAR-T cells was approximately 50% even at a low E:T ratio of 0.5:1, which was five times that of UNT cells. However, the killing rate of T3/28t CAR-T cells was similar to that of 19BBz, but their killing ability was obviously lower than that of T3/28 CAR-T cells at each ratio. These data indicate that the T3/28 chimera augments CAR-T cytotoxicity.

Consistent with the antigen-specific cytotoxicity of CAR-T cells, we detected increased levels of IFN-γ and granzyme B in supernatants in the presence of CAR-T cells but not UNT cells. In addition, compared with 19BBz CAR-T cells, the secretion of these effectors was significantly upregulated in the T3/28 CAR-T cells at different E:T ratios (figure 1B–D), which is consistent with the improved killing ability of T3/28 CAR-T cells as compared with 19BBz and T3/28t CAR-T cells. IL-17, secreted from both CD4^+^ and CD8^+^ T cells, acts synergistically with TNF-α.^21 22^ The amounts of IL-17, IL-2, perforin, and TNF-α released by T3/28 CAR-T cells were much higher than those released by the control groups (online supplemental figure S2A–C). Degranulation is a requisite process for perforin-granzyme-mediated killing. We found that CD107a was extraordinarily highly expressed on the surface of T3/28 CAR-T cells in response to Daudi cells (figure 1E), which was further confirmed in both CD4^+^ and CD8^+^ T cell subsets (online supplemental figure S2D).

**Figure 2:**
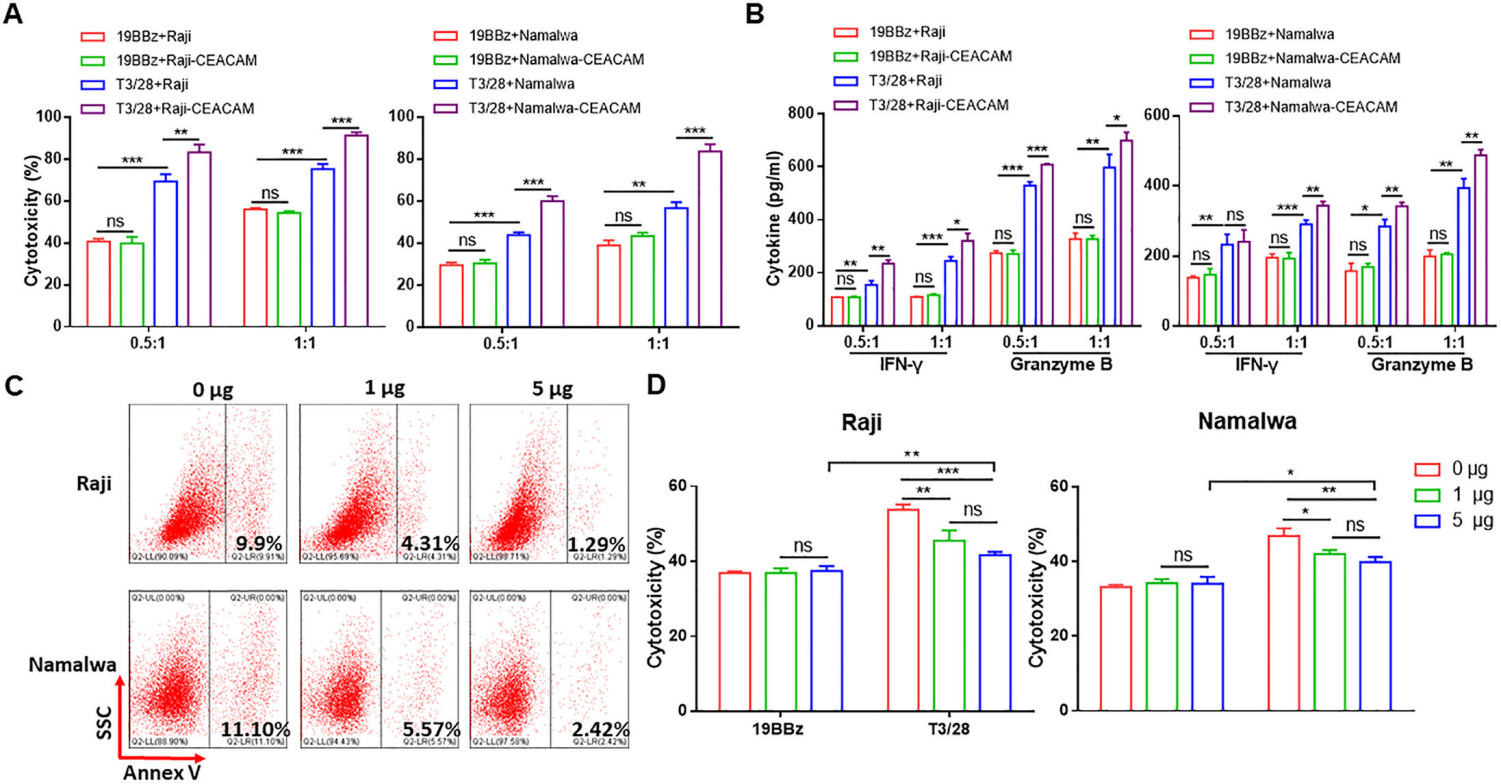
Switch receptor T3/28 enhances the cytotoxicity of 19BBz CAR-T cells depending on TIM-3 ligands. (A) The cytotoxicity of CAR-T cells against Raji-CEACAM1 and Namalwa-CEACAM1 cells was evaluated. (B) CAR-T cells and target cells (1×10^5^) were co-cultured for 18 hours at E:T ratios of 0.5:1 and 1:1. IFN-γ and granzyme B released by CAR-T cells co-cultured with Raji-CEACAM1 and Namalwa-CEACAM1 were detected using ELISA kit. (C) Flow cytometry was performed to detect PtdSer. The tumor cells were blocked by different amount of TIM-3 Fc chimera at 0, 1, and 5 μg mL^−1^ for 1 hour at RT, and then co-incubated with Annexin V. (D) The cytotoxicity of 19BBz and T3/28 CAR-T cells against tumor cells. Target cells and CAR-T cells were co-incubated with different amounts of TIM-3 Fc chimera for 1 hour; subsequently, the tumor cells were co-cultured with CAR-T cells for 8 hours at E:T ratio of 1:1. Data presented are the mean±SD of three separate experiments. ns means no significant difference, *p＜0.05, **p＜0.01, ***p＜0.001 compared with indicated group. CAR, chimeric antigen receptor; E:T, effector-to-target; IFN, interferon; PtdSer, phosphatidylserine; RT, room temperature; TIM-3: T-cell immunoglobulin mucin domain molecule 3.

To further assay the specificity of the killing activities of CD19 CAR-T cells, K562 cells were transformed to stably express CD19 (K562-CD19) (online supplemental figure S2E). The results indicated that the killing ability of T3/28 CAR- T cells against K562-CD19 cells was substantially higher than that of control groups, and no differences were observed in the cytotoxicity of T3/28 and 19BBz CAR-T cells against K562 cells (figure 1F). Similarly, co-culturing with K562-CD19 cells induced T3/28 CAR-T cells to secrete higher levels of IFN-γ, granzyme B, perforin, and TNF-α than those in the control group (online supplemental figure S2F), whereas there was no statistical difference between the T3/28 and 19BBz groups when co-cultured with K562 cells (online supplemental figure S2G).

### Switch receptor T3/28 enhances the cytotoxic ability of 19BBz CAR-T cells depending on TIM-3 ligands

Since the T3/28 chimera enhanced CAR-T cell cytotoxicity and cytokine secretion, we sought to determine whether T3/28 functional enhancement was dependent on TIM-3 ligands on tumor cells. The result of flow cytometry showed that the tumor cell lines expressed low levels of TIM-3 ligands, including Gal-9, CEACAM1, and medium levels of PtdSer in vitro (online supplemental figure S3A–D). The qPCR and ELISA results showed that the cell lines expressed medium levels of Gal-9 compared with HepG2 which highly expressed Gal-9,^23^ CEACAM1^24^ and HMGB1,^25^ especially Daudi and Raji cell lines (online supplemental figure S3E and F). Therefore, the tumor cell lines were transduced with a construct encoding one of the TIM-3 ligands, CEACAM1 (online supplemental figure S3G). As shown in figure 2A, dose-dependent killing was observed in T3/28 CAR-T cells co-cultured with CEACAM1 overexpressing Raji (Raji-CEACAM1) or Namalwa (Namalwa-CEACAM1) cells at E:T ratios of 0.5:1 and 1:1 (figure 2A). Killing of 19BBz against parent tumor cells and CEACAM1 overexpressing tumor cells was similar, but T3/28 CAR-T cells showed evidently higher killing against CEACAM1 overexpressing tumor cells at each ratio as compared with parent tumor cells (figure 2A). Consistently, the levels of cytokines from 19BBz CAR-T cells stimulated with parent tumor cells or CEACAM1 overexpressing tumor cells were similar, while the T3/28 CAR-T cells secreted higher levels of cytokines when co-cultured with CEACAM1 overexpressing tumor cells compared with parent tumor cells (figure 2B).

To further determine whether T3/28 enhances the cytotoxicity of 19BBz CAR-T cells in a TIM-3 ligand-dependent manner, recombinant human TIM-3 Fc chimera was introduced to block the TIM-3 ligand in vitro. TIM-3 ligand PtdSer expressed on Raji and Namalwa cells was successfully blocked by the TIM-3 Fc chimera (figure 2C). The TIM-3 Fc chimera significantly downregulated the cytotoxicity of T3/28 CAR-T cells against target cells, while the cytotoxicity of 19BBz CAR-T cells was not affected (figure 2D), demonstrating that the enhanced cytotoxicity of T3/28 CAR-T cells depends on TIM-3 ligands.

### T3/28 CAR-T cells expanded ex vivo maintain a superior phenotype

Generally, a less-differentiated phenotype can promote T cell survival and antitumor activity. Therefore, we determined the possible mechanisms underlying the higher incidence of cytotoxicity associated with T3/28 CAR-T cells than with 19BBz CAR-T cells. After co-culturing with tumor cells, T3/28, 19BBz, and UNT cells were stimulated until 18 days, and activation, differentiation, exhaustion and apoptosis status were evaluated at the indicated time points. The results indicated that T3/28 CAR-T cells maintained a superior phenotype.

**Figure 3:**
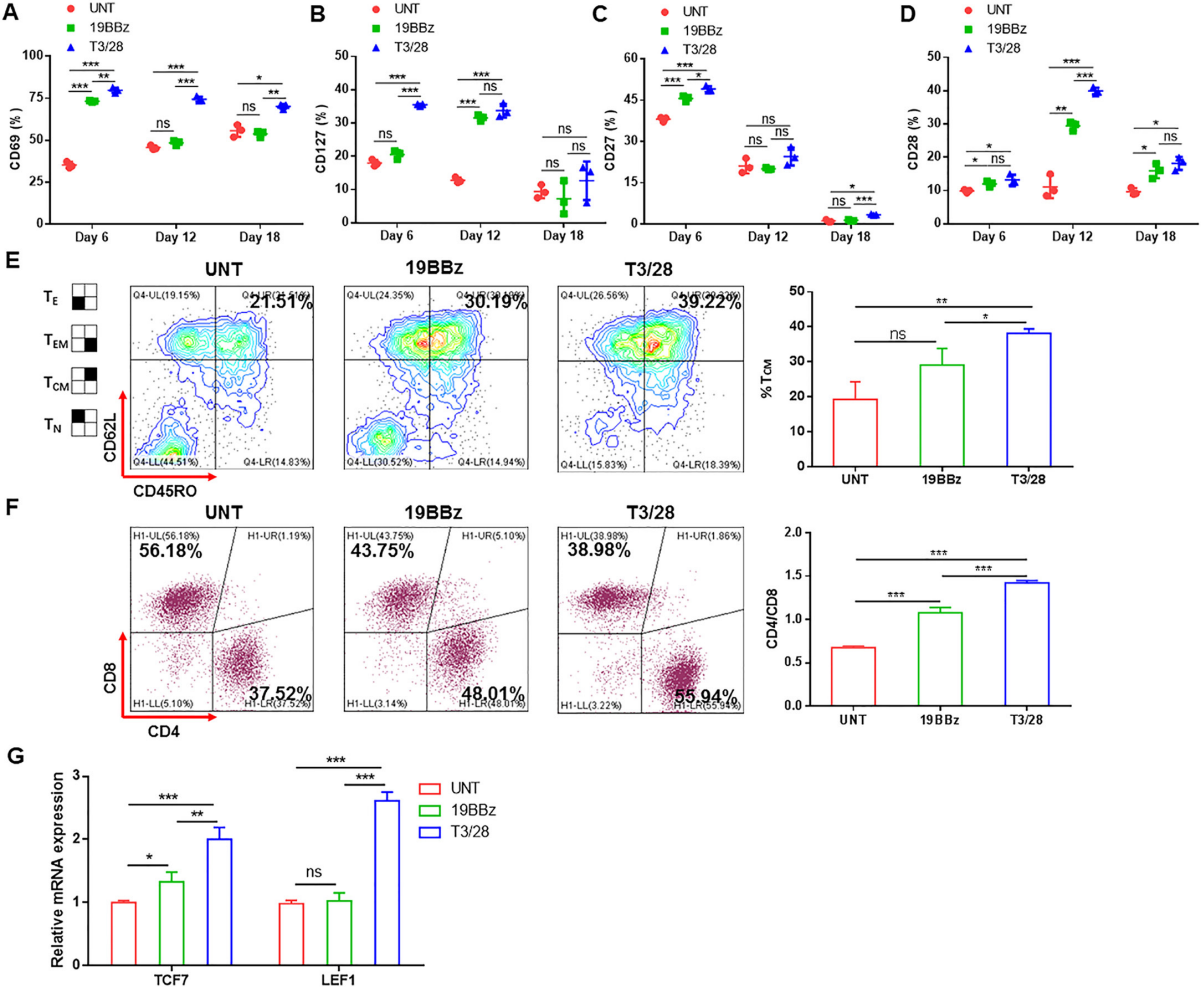
T3/28 CAR-T cells expanded ex vivo and maintain a superior phenotype. Flow cytometry was performed to detect expression of activation markers CD69 (A) and CD127 (B) and low-differentiation associated costimulatory molecules CD27 (C) and CD28 (D) on T cells stimulated with tumor cells. T3/28 CAR-T cells, 19BBz CAR-T cells, and UNT cells were expanded until day 18, and activation and differentiation markers were evaluated at indicated time points. (E) The T_CM_ compartment, with a CD45RO^+^CD62L^+^ phenotype, was assayed using flow cytometry. (F) CD4:CD8 ratio of CAR-T cells stimulated with tumor cells at day 9 post transduction was evaluated using flow cytometry. (G) Quantitative PCR was used to analyze memory stem-like-associated transcription factors TCF7 and LEF1 in CAR-T and control cells. Data presented are the mean±SD of three separate experiments. ns means no significant difference, *p＜0.05, **p＜0.01, ***p＜0.001 compared with indicated group. CAR, chimeric antigen receptor; mRNA, messenger RNA; T_CM_, central memory T; T_E_, effector T; T_EM_, effector memory T; T_N_, naive T; UNT, untreated T cells.

The expression of activation markers CD69 and CD127 on T3/28 CAR-T cells was enhanced compared with that on 19BBz CAR-T cells (figure 3A, B). Higher levels of low-differentiation associated costimulatory molecules CD27 and CD28^26^ were expressed in T3/28 CAR-T cells than in controls (figure 3C, D). T cells can be divided into four phenotypes according to CD62L and CD45RO.^27^ Naive and central memory T (T_CM_) cells have a higher proliferative capacity and are less-differentiated than effector memory T (T_EM_) cells. To evaluate the potential advantages of T cells undergoing prolonged stimulation with tumor cells, the defined subsets were assayed using flow cytometry. Indeed, 19BBz CAR-T cells preferentially enriched the T_EM_ cells, while activated T3/28 CAR-T cells appeared to enrich the T_CM_ compartment (figure 3E). Further qPCR analysis demonstrated an upregulation of key memory stem-like-associated transcription factors (ie, *LEF1* and *TCF7*) in T3/28 CAR-T cells than in 19BBz CAR-T cells and UNT cells (figure 3G). CD8^+^ CAR-T cells exhibit robust short-term effector function but become rapidly exhausted, while CD4^+^ CAR-T cells persist after tumor challenge and sustain their effector potency.^27^ In addition, CD4^+^ T cells, which play an essential role in antitumor response, have also been observed to demonstrate antitumor activity in preclinical models of hematological and solid tumors.^28 29^ We compared the CD4:CD8 ratio of CAR-T cells stimulated with tumor cells at day 9 post transduction and found a significantly higher percentage of CD4^+^ CAR-T cells in the T3/28 group than in the 19BBz CAR-T cells, suggesting that T3/28 CAR-T cells are prone to have persistent antitumor potency (figure 3F).

These data thus confirm that T3/28 CAR-T cells retain superior phenotype characteristics compared with their control counterparts, which might contribute to endowing T3/28 CAR-T cells with high cytotoxicity against target cells.

### Switch receptor T3/28 inhibits CAR-T cell exhaustion

Exhaustion affects CAR-T cell function and persistence, which is a barrier to effective CAR-T cell response.^30^ We characterized surface markers indicative of T-cell exhaustion. We found that the T3/28 chimera downregulated the expression of several immune checkpoints associated with exhausted T cells (eg, PD-1, TIGIT, and LAG-3) (online supplemental figure S4A), demonstrating the favorable effects of the switch receptor T3/28 on mitigating exhaustion in CAR-T cells. Further, qPCR analysis demonstrated a downregulation of key exhaustion-associated transcription factors (ie, *Blimp-1* and *T-bet*) in T3/28 CAR-T cells as compared with 19BBz CAR-T cells and UNT cells (online supplemental figure S4B). In addition, lower levels of the exhaustion-related TOX family genes (TOX, TOX2, TOX3, TOX4) were detected in T3/28 CAR-T cells than in 19BBz CAR-T cells and UNT cells (online supplemental figure S4C). As shown in online supplemental figure S4D, no difference in regulatory T cell (Treg) subsets, a suppressor of antitumor immunity, was found between the two CAR-T groups, suggesting that the switch receptor T3/28 has no significant effect on Treg cell differentiation in CD19 CAR-T cells. Taken together, these data demonstrated that CAR-T cells modified with T3/28 chimeras were programmed to sustain a less-exhausted phenotype.

**Figure 4:**
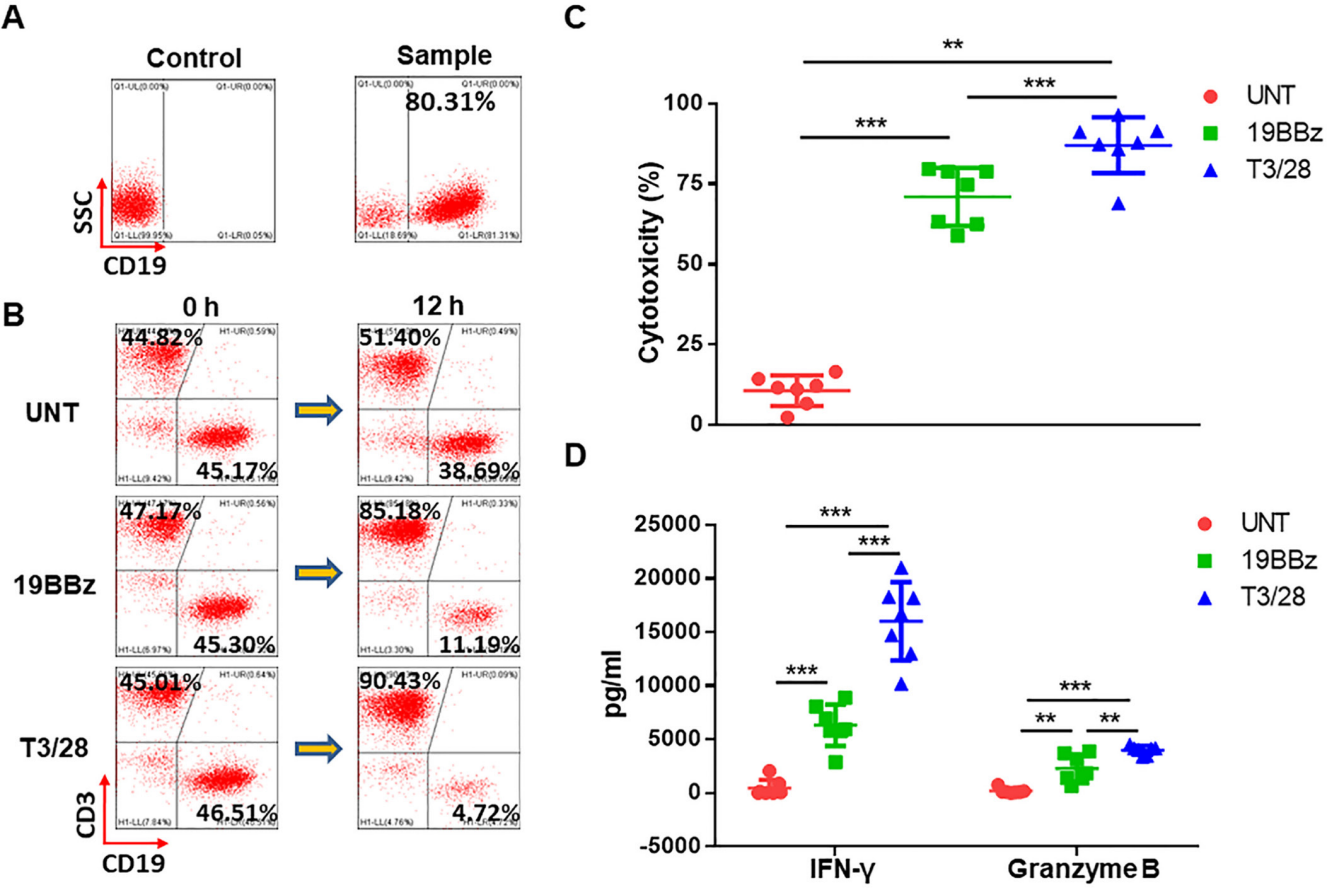
T3/28 CD19 CAR-T cells effectively eliminate clinically derived B lymphoma cells. (A) Clinically derived samples highly expressing CD19 antigen from several patients. (B) B lymphoma cells were mixed with effector cells followed by staining with anti-CD3 and anti-CD19 antibodies, and 12 hours later detection was performed by a flow cytometer. (C) B lymphoma cells (1×10^5^) were prepared in cell culture medium, then B lymphoma cells were co-incubated with CAR-T cells for 12 hours at E:T ratio of 1:1 in a total volume of 500 µL. The cytotoxicity of UNT, 19BBz, and T3/28 CAR-T cells was evaluated by LDH release assay. (D) IFN-γ and granzyme B released by T cells co-cultured with B lymphoma cells were assayed using ELISA kit. Data presented are the mean±SD of three separate experiments. **p＜0.01, ***p＜0.001 compared with indicated group at the same E:T ratio. CAR, chimeric antigen receptor; E:T, effector-to-target; IFN, interferon; LDH, lactate dehydrogenase; UNT, untreated T cells.

**Table 1:** Patient characteristics

	Sex	Age	Disease type	Treatment	Therapeutic regimen
Pt1	Female	76	B-ALL	No	
Pt2	Female	52	B-ALL	No	
Pt3	Male	68	CLL	No	
Pt4	Male	75	DLBCL	No	
Pt5	Male	72	DLBCL	No	
Pt6	Male	79	B-ALL	No	
Pt7	Male	56	DLBCL	Yes	R-CHOP

ALL, Acute lymphoblastic leukemia; CLL, Chronic lymphocytic leukemia; DLBCL, Diffuse large B cell lymphoma; R-CDOP, Rituximab, cyclophosphamide, doxorubicin, vincristine, prednisone.

### T3/28 CD19 CAR-T cells possess superior cytotoxicity against clinically derived B cell lymphoma

To assess the cytotoxicity of T3/28 CAR-T cells against primary B lymphoma, several samples highly expressing the CD19 antigen from untreated or treated patients were included (figure 4A, table 1). We set up co-culture systems of B lymphoma with effector CAR-T cells from three donors at an E:T ratio of 1:1 to test the CAR-T cell cytotoxicity to clinically derived B lymphoma. After co-culture, T cells and residual target cells were analyzed using flow cytometry, and we found an obvious decrease in target cells following co-culture with T3/28 CAR-T cells compared with 19BBz CAR-T cells (figure 4B). The LDH release assay revealed that CD19 CAR-T cells mediated efficient lysis of both B cell acute lymphoid leukemia and chronic lymphocytic leukemia, while UNT cells showed no effect. Moreover, improved killing ability of T3/28 CAR-T cells against primary B lymphoma was observed in all samples (figure 4C), which was consistent with the results shown in figure 1. In addition, the secretion of effectors (ie, IFN-γ and granzyme B) was significantly upregulated in the T3/28 CAR-T cells than in the 19BBz CAR-T cells (figure 4D). These data indicated the superior cytotoxicity of T3/28 CAR-T cells and their potential application in B lymphoma therapy.

### Switch receptor T3/28 mediates superior antitumor cytotoxicity in a xenograft model

**Figure 5:**
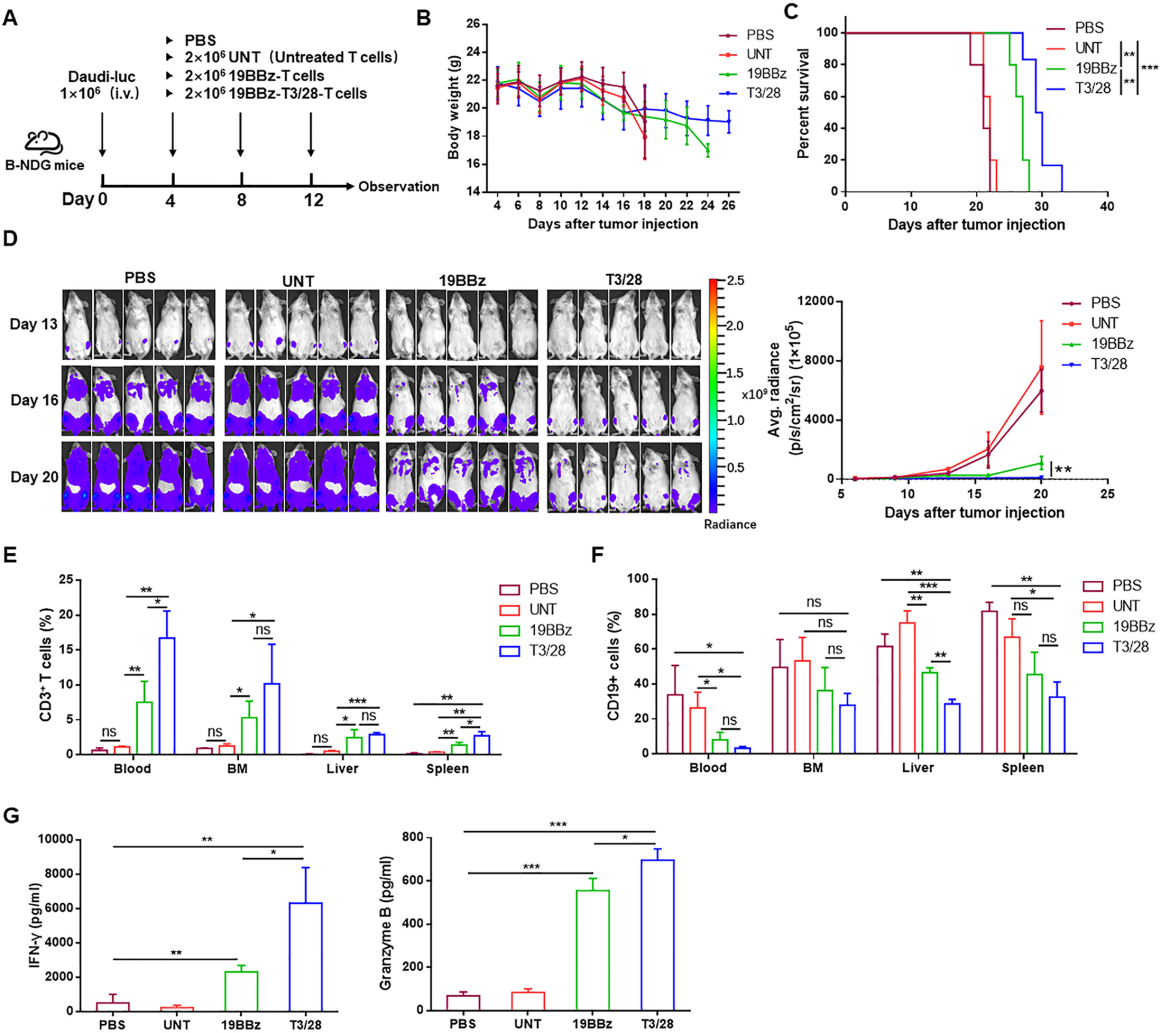
Switch receptor T3/28 mediates superior antitumor cytotoxicity in vivo. (A) The scheme of experimental design. (B) Body weights of the mice were measured two to three times per week. The values are presented as the mean±SE of the mean. (C) Survival was evaluated from the first day of tumor cell injection until death. Statistical analysis was performed using the log-rank (Mantel-Cox) text. **p＜0.01, ***p＜0.001 compared with indicated group. (D) Tumor burden was determined by weekly bioluminescent imaging (BLI) (n=5 mice per group). The T cells (E) and Daudi-luc cells (F) from visceral organs (BM (bone marrow), liver, spleen, and kidney were analyzed on a flow cytometer. (G) Serum levels of granzyme B and IFN-γ was determined using ELISA kit. Data presented are the mean±SD of three separate experiments. ns means no significant difference, *p＜0.05, **p＜0.01, ***p＜0.001 compared with indicated group. IFN, interferon; PBS, phosphatic buffer solution.

Since CAR-T therapy may cause on-target off-tumor side effects, it is ideal to reduce the toxicity by increasing the specificity of multiple tumor markers. In this regard, the novel T3/28 chimera, possibly targeting PtdSer, Gal-9, and CEACAM1 on the tumor cells, was developed to synergistically enhance the ability of engineered T cells to kill B lymphoma. Further, we compared the antitumor activity of T3/28 CAR-T, 19BBz CAR-T, and UNT cells in preclinical models. For the in vivo validation, the Fluc-transduced Daudi tumor cell line was inoculated intravenously into B-NDG mice to establish a B lymphoma model. Four days after tumor cell injection, the mice received CAR-T or UNT cells (figure 5A). Changes in body weight of the mice were closely monitored during the experiment, and the results indicated that there were no obvious differences among the various groups (figure 5B). As shown in figure 5C, D, T3/28 CAR-T cells significantly controlled tumor growth and extended overall survival. The median survival times of mice in the PBS, UNT, 19BBz, and T3/28 groups were 21, 21.8, 26.6, and 29.8 days, respectively.

In the xenograft models using the Burkitt’s lymphoma cell line Daudi, T3/28 CAR-T cells showed superior antitumor activity. To further evaluate CAR-T cell expansion in vivo, the visceral organs (bone marrow, liver, and spleen) and peripheral blood from mice were harvested simultaneously and cell populations were analyzed. The data showed that circulating T3/28 CAR-T cells were numerically more abundant than 19BBz CAR-T cells, as well as CD4^+^ and CD8^+^ T cells. Compared with the 19BBz group, more CAR-T cells were also detected in the spleen, and more CD8^+^ T cells were measured in the liver in the T3/28 group (figure 5E, online supplemental figure S5A and B). Moreover, CD25 and CD27 expression in CAR-T cells was increased in the bone marrow and spleen, and lower levels of TIGIT and LAG3 expression in CAR-T cells were found in the spleen of the T3/28 group (online supplemental figure S5C and D). At the same time, the CD19^+^ Daudi-Fluc cells from visceral organs (bone marrow, liver, spleen, and kidney) were analyzed on a flow cytometer, and the T3/28 group presented significantly lower levels of CD19^+^ cells than the PBS and UNT groups. More importantly, less CD19^+^ Daudi cell infiltration was found in the liver in the T3/28 group compared with the 19BBz group (figure 5F). Meanwhile, T3/28 CAR-T cells released higher levels of IFN-γ and granzyme B than those released by the control group (figure 5G). In addition, the spleen and kidney weight/body weight ratios in the PBS and UNT groups were higher than those in the CAR-T group (online supplemental figure S5E and F), which might be attributed to the infiltration of Daudi cells. Collectively, the in vivo data demonstrated that T3/28 CAR-T cells showed potent antitumor activity in mice.

To further evaluate the broad effect of the switch receptor T3/28 on CAR-T cells, another CAR targeting CD138 was designed (online supplemental figure S6A). Improved killing ability of 138-T3/28 CAR-T cells against multiple myeloma cell lines, as compared with 138BBz CAR-T cells, was observed in vitro (online supplemental figure S6B). In a murine model of subcutaneous RPMI8226, when compared with control mice receiving 138BBz CAR-T cells, 138-T3/28 CAR-T cell treatment significantly lead to reduction in tumor burden and improvement in survival (online supplemental figure S6C-F). In addition, a significant increase in CAR-T cell infiltration was observed in tumors from mice treated with intravenous injection of 138-T3/28 CAR-T cells (online supplemental figure S6G).

### T3/28 CD19 CAR-T cells caused no detectable CRS or evident lesions

CRS and neurotoxicity caused by high concentrations of serum cytokines are the most commonly observed and expected life-threatening complications following CAR-T cell infusion.^31^ Serum cytokines, including IFN-γ, IL-6, IL-8, MCP-1, IL-1β and so on contribute to CRS.^32 33^ In this study, CRS toxicity was observed in accordance with clinical classification.^31 34^ For example, in grade 1, symptoms are not life threatening, such as fever, nausea, headache, myalgia, malaise, or fatigue; grade 2 includes symptoms that require and respond to intravenous fluids or low-dose vasopressors, such as grade 2 organ toxicity or fraction of inspired oxygen less than 40%.

To investigate the potential toxicities of T3/28 CAR-T cells in immunodeficient mice that favor the expansion of adoptively transferred T cells, we set up a mouse model with Daudi cells. Four days later, UNT, 19BBz, or T3/28 CAR-T cells were intravenously infused into tumor-bearing mice for three times (online supplemental figure S7A). After each infusion, no detectable negative symptoms, including anepithymia and malaise, were observed. Mice were monitored for approximately 2 weeks and then euthanized to assess organ toxicity. Although mice treated with T3/28 CAR-T cells showed moderately higher levels of IL-2, IL-8, and TNF-α compared with the 19BBz CAR-T cell group, no significant changes were found in other cytokines (online supplemental figure S7B), suggesting that T3/28 CAR-T cells promote tumor control (figure 4) in tumor-bearing B-NDG mice without causing detectable organ toxicity. Similarly, no evident lesions were observed in the tissue sections (online supplemental figure S7C).

### T3/28 CAR-T cells possess more potent persistence ability in vivo

It has been reported that second-generation CD28-based CAR-T cells lack durable persistence in responding patients compared with the persistence observed with 4-1BB-containing CARs. Therefore, we constructed a second CAR based on 4-1BB (online supplemental figure S1A). Diverse factors affect the persistence of CAR-T cells, such as scFv,^35^ costimulatory signals,^36^ and spacers,^37^ but little is known about the impact of the switch receptor on the persistence of CAR-T cells. To further assess the enhanced antitumor response of T3/28 CAR-T cells, we evaluated the persistence of CAR-T cells in vitro and in vivo. First, we assessed the apoptosis and proliferation of CAR-T cells. The results showed that T3/28 CAR-T cells expressed significantly less active caspase-3 compared with 19BBz CAR-T cells (online supplemental figure S8A). In line with this finding, the anti-apoptotic molecules Bcl-2 and Bcl-xl were upregulated in T3/28 CAR-T cells at day 12 post transduction compared with controls (online supplemental figure S8B), further confirming that the switch receptor T3/28 exerts an anti-apoptotic effect on T cells. In addition, the ratio of CAR-positive T cells in T cell subsets was analyzed over time. We found that the ratios of CAR-positive T cells increased consistently over the first 3 weeks before decreasing to some extent, illustrating that the vitality of CAR-T cells was maintained for approximately 20 days, and exhausted soon afterwards (online supplemental figure S8C). Consistently, T3/28 CAR-T cells maintained a higher proportion of T cells and exhibited a more distinct proliferation phenotype as compared with 19BBz CAR-T cells (online supplemental figure S8D and E), which was consistent with a steep proliferation curve of T3/28 cells after incubation with target cells (online supplemental figure S8F).

**Figure 6:**
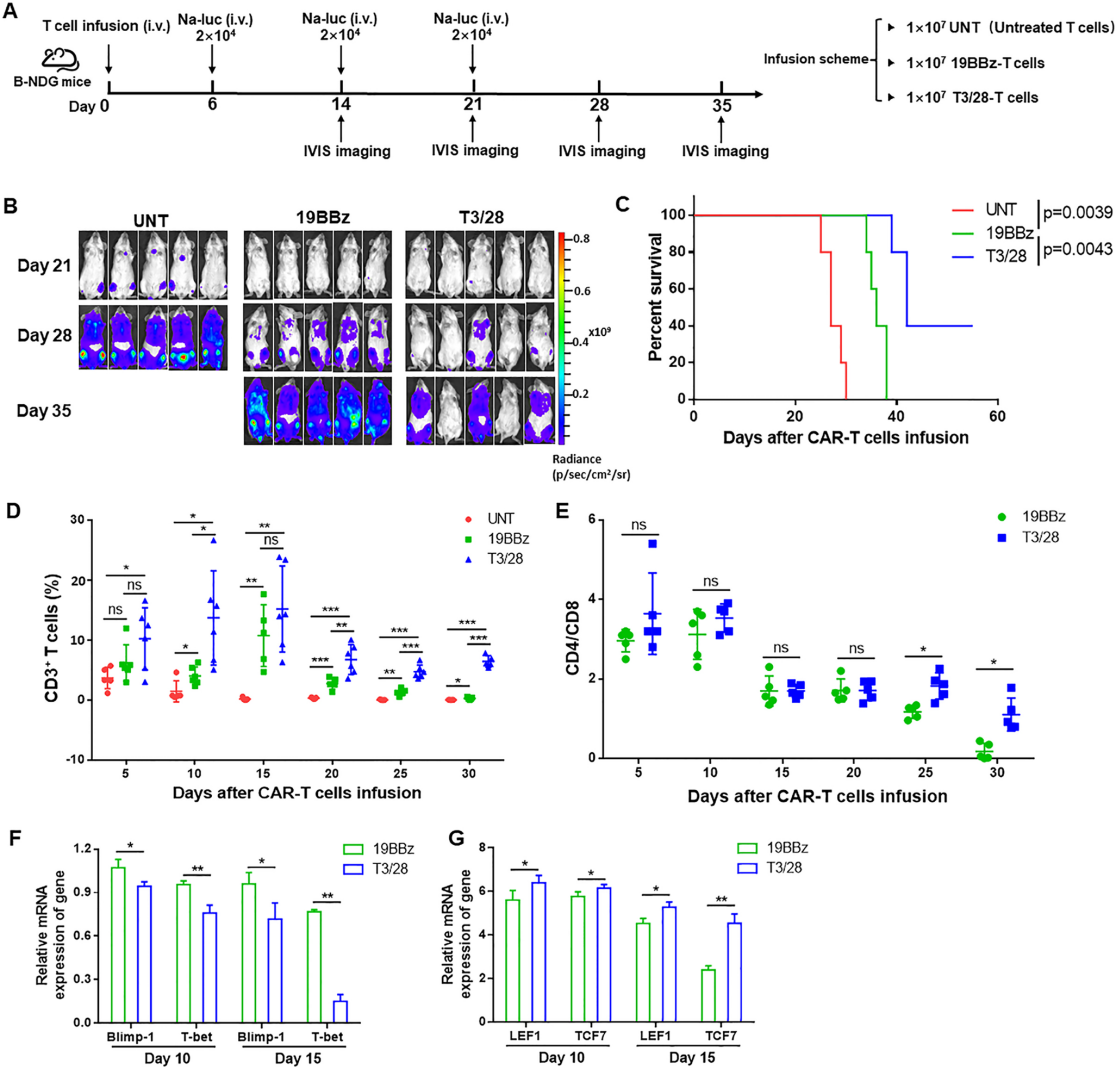
T3/28 CAR-T cells possess more potent persistence ability in vivo. (A) The scheme of experimental design. (B) Tumor burden was determined by bioluminescent imaging (n=5 mice per group). (C) Survival was evaluated from the first day of tumor injection until death. Statistical analysis was performed using the log-rank (Mantel-Cox) text. (D) The UNT and CAR-T cells from blood were monitored every 5 days. (E) The change of CD4: CD8 ratio was monitored every 5 days. Quantitative PCR analysis of exhaustion associated transcription factors (Blimp-1 and T-bet) (F) and memory stem-like-associated transcription factors (LEF1 and TCF-7) (G) on 19BBz and T3/28 CAR-T cells was performed. Data presented are the mean±SD of three separate experiments. ns means no significant difference, *p＜0.05, **p＜0.01, ***p＜0.001 compared with indicated group. CAR, chimeric antigen receptor; i.v., intravenously; IVIS, In Vivo Imaging System.

To test whether the T3/28 chimera could enhance the persistent antitumor ability of CD19 CAR-T cells in vivo, CAR-T cells were intravenously injected into B-NDG mice followed by three tumor challenges (figure 6A). Tumor growth was monitored by measuring changes in tumor bioluminescence over time. Tumor bioluminescence increased rapidly in the mice treated with control UNT cells. On days 21 and 28 post CAR-T cell injection, substantial differences were observed in tumor burden among the three groups, and tumor growth was significantly controlled by T3/28 CAR-T cells (figure 6B). The persistence of CAR-T cells is tightly correlated with the durability of remission in mice. Infusion of T3/28 CAR-T cells led to improved survival of tumor-bearing mice compared with 19BBz CAR-T cells and control UNT cells (figure 6C). However, no significant changes in the body weight of the mice were observed among the various groups (online supplemental figure S9A).

Based on the above findings, we hypothesized that T3/28 CAR-T cells may be able to eliminate Na-luc cells constantly because of their superior proliferative ability in vivo. To explore this possibility, we performed flow cytometry to quantify CAR-T cells in the blood at the indicated time points. As expected, significantly greater absolute numbers of T3/28 CAR-T cells than of 19BBz CAR-T cells were observed in the blood until 30 days after T-cell infusion (figure 6D), accompanied by more CD4^+^ and CD8^+^T cells in the T3/28 group (online supplemental figure S9B and C). Moreover, we found an obviously higher CD4/CD8 ratio of CAR-T cells in the T3/28 group than in the 19BBz group after 20 days and 30 days (figure 6E). Along with the T3/28 CAR-T cells showing strong, quick, and persistent amplification, the peak of CAR-T cell expansion reached its maximum at 10 days after CAR-T cell infusion. Three mice in the T3/28 group possessed moderate numbers of CAR-T cells until 35 days after T-cell infusion (online supplemental figure S9D). In addition, expression of the exhaustion-associated transcription factors *Blimp-1* and *T-bet* in CAR-T cells was significantly decreased in the T3/28 group (figure 6F). Consistent with the in vitro findings, T3/28 CAR-T cells from the blood showed significantly higher levels of memory stem-like-associated transcription factors *LEF1* and *TCF7* than the 19BBz group (figure 6G). Additionally, we observed a stronger persistence ability of CD138-T3/28 CAR-T cells compared with 138BBz CAR-T cells in another tumor-challenge model (online supplemental figure S10A). CD138-T3/28 CAR-T cells significantly improved the survival of tumor-bearing mice compared with that of 138BBz CAR-T cells (online supplemental figure S10B), and there was an obviously higher proportion of CD138-T3/28 CAR-T cells in the blood (online supplemental figure S10C). Together, these results indicate that T3/28 CAR-T cells mediate enhanced antitumor responses and expand more robustly relative to traditional 4-1BB CAR-T cells.

### IL-21/Stat3 axis contributes to enhanced cytotoxicity of T3/28 CAR-T cells

**Figure 7:**
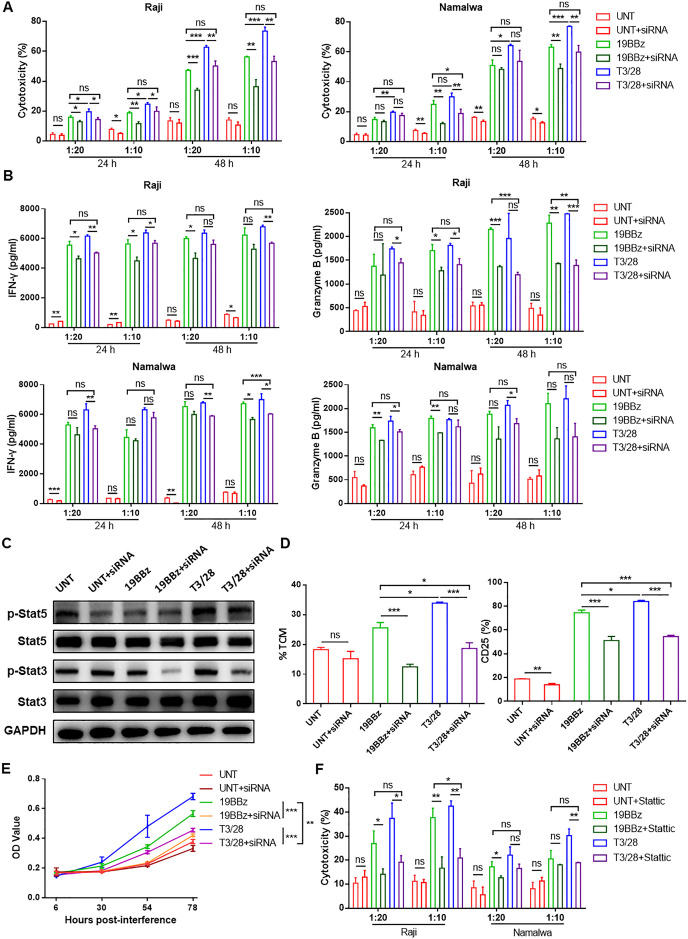
IL-21/Stat3 axis contributes to enhanced cytotoxicity of T3/28 CAR-T cells. (A) At 24 hours and 48 hours of co-culture at lower E:T ratios of 1:20 and 1:10, the cytotoxicity of CAR-T cells with or without IL-21 siRNA targeting B lymphoma cell lines (Raji and Namalwa) was analyzed using LDH assay kit. (B) After co-culture for 24 hours and 48 hours, the supernatants were harvested and an assay was performed to measure IFN-γ and granzyme B release by T cells with or without IL-21 siRNA co-cultured with Raji and Namalwa cells using ELISA kit. (C) After antigen stimulation, downstream signaling of IL-21 was altered as determined by measuring Stat3/5 phosphorylation using western blot. (D) After antigen stimulation, changes in T_CM_ subpopulations and CD25 expression on UNT, 19BBz and T3/28 CAR-T cells with or without IL-21 siRNA were analyzed. (E) The proliferation ability of T3/28 CAR-T cells was obviously affected with IL-21 knockdown. The statistical difference is presented at 78 hours only. (F) At 24 hours and 48 hours of co-culture at lower E:T ratios of 1:20 and 1:10, the cytotoxicity of CAR-T cells, with or without Stat3 inhibitor (static), targeting B lymphoma cell lines (Raji and Namalwa) was analyzed using LDH assay kit. Data presented are the mean±SD of three separate experiments. ns means no significant difference, *p＜0.05, **p＜0.01, ***p＜0.001 compared with indicated group. CAR, chimeric antigen receptor; E:T, effector-to-target; IFN, interferon; IL, interleukin; LDH, lactate dehydrogenase; OD, optical density; siRNA, small interferring RNA; T_CM_, central memory T; UNT, untreated T cells.

To clarify the mechanism responsible for the enhanced cytotoxicity of T3/28 CAR-T cells, RNA-Seq analysis was performed using T3/28 CAR-T cells and 19BBz CAR-T cells. The heat map showed that proliferation-associated genes, including IL-21, were significantly higher in T3/28 CAR-T cells (online supplemental figure S11A). IL-21, produced predominantly by activated CD4^+^ T cells, has been reported to promote T cell-mediated tumor rejection and has pleiotropic effects on immunity via the IL-21 receptor. Markley and Sadelain reported that CAR-T cells engineered to express IL-21 efficiently eliminated tumor cells with long-term persistence in vivo.^38^ The IL-4/21 inverted cytokine receptor maintains CAR-T cell survival, attenuates exhaustion, and improves CAR-T cell potency in a suppressive solid-tumor immune microenvironment through the IL-21/Stat3 axis.^39^ In addition, the novel CAR construct containing the YXXQ motif of the IL-21 receptor, which activates Stat3 signaling, mediates superior antitumor effects in vivo, which is essential for CAR-T cell persistence.^40^ Therefore, we speculated that IL-21 might contribute to the enhanced cytotoxicity and persistence of T3/28 CAR-T cells. qPCR and ELISA results indicated that the expression of IL-21 in T3/28 CAR-T cells was higher than that in the other groups. Next, IL-21 was knocked down to determine its role in the cytotoxicity of CAR-T cells (online supplemental figure S11B and C). We found that there was no difference between the CAR-T and CAR-T-interference groups at higher E:T ratios of 0.5:1 and 1:1 (data not shown). However, at 24 hours and 48 hours of co-culture at lower E:T ratios, we found that the killing rate of IL-21 siRNA groups was approximately 10% lower than that of T3/28 groups, and the killing rates of T3/28 CAR-T cells after IL-21 interference showed no significant difference with the 19BBz group (figure 7A), and a similar trend was found for IFN-γ and granzyme B in these groups (figure 7B). Stat3, a hallmark of IL-21 signaling, is considered to be the major transcription factor responsible for IL-21 mediated effects, and 40% of genes are regulated by IL-21.^41^ Stat5 phosphorylation is reported to transiently occur in IL-21 signaling, whereas the activation of Stat3 is more sustained.^42^ As shown in figure 7C, in the presence of IL-21 siRNA, Stat3 phosphorylation was greatly decreased in T3/28 and 19BBz CAR-T cells, accompanied by a weak change in Stat5 phosphorylation. To evaluate the differentiation phenotype of T3/28 CAR-T cells undergoing interference with IL-21 siRNA, the defined subset composition was assayed using flow cytometry. The results showed that the T_CM_ subpopulations were downregulated in T3/28 CAR-T cells undergoing IL-21 interference (figure 7D, left panel). After in vitro antigen stimulation, T3/28 CAR-T cells maintained a higher proportion of T cells expressing CD25 than the interference groups (figure 7D, right panel). Without antigen stimulation in vitro, the T3/28 CAR-T cells with IL-21 interference showed significantly weaker proliferation than the control CAR-T cells (figure 7E). Consistent with the above results, treatment with the Stat3 inhibitor Stattic weakened the toxicity of T3/28 CAR-T cells (figure 7F). These results suggest a key role of the IL-21/Stat3 axis in supporting the cytotoxicity and persistence of CAR-T cells.

## Discussion

Taken together, the data presented in this work provide several insights relevant for a chimeric switch receptor T3/28 linked to the second CAR to overcome the hurdles of poor CAR-T cell persistence. Using different approaches, our data demonstrate that the switch receptor T3/28 endows the CAR-T cell with a superior phenotype and enhances CAR-T cytotoxicity and persistence in vitro and in vivo. Compared with controls, the T3/28 CAR-T cells showed complete tumor control following tumor re-challenge but without causing detectable side effects, including CRS or neurotoxicity. Commonly, the risk factors for CRS and neurotoxicity are closely related to the dose of CAR-T cell infusion.^43^ Although the optimal dose and re-dosing schedule of CAR-T cells for cancers is undefined, there was no detectable toxicity in response to a high-dose of CAR-T cells in this study. We further confirmed that the switch receptor T3/28 promotes CD19 CAR-T cytotoxicity via the IL-21/Stat3 axis.

We chose CD19 as the target for our studies, since satisfactory clinical efficacy has been shown by CD19 CAR-T cells against B-lineage lymphomas. Recent studies have reported that the development of persistence characteristics is associated with increased antitumor efficacy in adoptively transferred T cell subsets. Although the traditional 19BBz CAR could mediate a strong antitumor response, the effect was only transient, further emphasizing the importance of in vivo persistence of chimeric switch receptors for effective and durable antitumor immunity. Guercio *et al* reported that the co-stimulatory domain CD28 incorporating the OX40 domain exhibits remarkable cytolytic activity ex vivo and in vivo against tumors, with sustained proliferation and pro-inflammatory cytokine production.^44^ Our CAR incorporated a T3/28 chimera that may confer prolonged persistence of CAR-T cells by preventing exhaustion through downstream CD28 signaling (figures 3A–D, G, 5E and 6D, F, G), which was consistent with the point that the CD28 domain is essential for CAR-T persistence in vivo.^44 45^

Because CD28 and 4-1BB signaling activate different pathways, combining them in a CAR may overcome the limitations of each individual co-stimulatory domain. It has been reported that the CD28 domain, which provides robust cell activation and expansion responses, conferred CAR-T cells resistance to TGF-β-mediated inhibition, making these cells more effective against tumors.^46^ In addition, Zhang reported that using a 19BBz CAR incorporated with multiple OX40 is superior to a tandem OX40.^47^ Thus, we speculate that using a T3/28 chimera should be beneficial to 4-1BB based CAR-T cells through further activating CD28 mediated signaling, and that 19BBz-T3/28 might be superior to 4-1BB/CD28-based third generation CARs to some extent. Interestingly, CTLA4-CD28 chimera gene modification can enhance the function of CAR-T cells and help rescue hypofunctional CAR-T cells.^8^ T cells transduced to express Tigit-28 chimera or PD1-28 chimera exhibited upregulation of activation markers and mediated superior antitumor cytotoxicity in a xenograft model.^8–11^ Moreover, in clinical trials, the application of the PD1-28 chimera presented encouraging results.^48^ The Carl June group verified that the co-stimulatory domain 41BB promotes memory T cells and CD28 promotes effector T cells. In this study, our data showed that T3-CD28 signaling combined with 41BB might benefit CAR-T cells to some extent. However, the signaling pathway involved in T3/28 chimera engineered CAR-T cells requires further investigation.

RNA sequence analysis revealed that IL-21 production was higher in T3/28 CAR-T cells than in 19BBz CAR-T cells with or without antigen stimulation (online supplemental figure S11). It has been reported that adoptive transfer of CAR-T cells cultured with IL-21 exhibited improved control of B cell malignancy in mice,^49^ and IL-21 interference led to decreased numeric expansion and functionality of CAR-T cells, suggesting that IL-21 signaling is essential for optimal CAR-T function. Consistently, in this study, IL-21 interference weakened the cytotoxicity of T3/28 CAR-T cells (figure 7A–B). Many studies have reported that IL-21 supports the development of memory CAR-T cells with superior antitumor activity,^50–52^ which mirrored the results of our study where we observed a higher proportion of T_CM_ in the T3/28 group (figures 3E and 7D), suggesting that IL-21 signaling might contribute to the persistence of T3/28 CAR-T cells. It is well known that IL-21 preferentially activates Stat3 through its association motif YXXQ within the IL-21 receptor.^42^ As expected, in the presence of an inhibitor of Stat3, T3/28 CAR-T cells showed decreased antitumor effects in vitro (figure 7F). Thus, the IL-21/Stat3 axis is essential for enhancing T3/28 CAR-T cell function. Since IL-21-mediated expansion of Vγ9Vδ2 T cells is limited by the TIM-3 pathway,^53^ and activation of Gal-9/TIM-3 signaling decreases IL-21 production,^54^ the TIM3-CD28 chimera might orchestrate IL-21/Stat3 axis in T3/28 CAR-T cells via an unknown mechanism.

We acknowledge a limitation of our study. It is well known that several ligands are responsible for TIM-3-mediated inhibition of T cell activation and proliferation. The first ligand attributed to TIM-3 function is Gal-9. The precise binding site for Gal-9 on TIM-3 has not been clearly defined, and its interaction suppresses immune responses.^55^ Recently, it was reported that Gal-9 regulates TIM-3 cell surface clustering, which is necessary for the suppressive function of TIM-3.^56^ As the most recently discovered ligand, CEACAM1, which is highly expressed by some tumor cells, plays a role in dampening T cell responses.^57 58^ As a functional TIM-3 ligand,^59^ PtdSer is an essential component of bilayer cell membranes and is normally present in the inner leaflet. However, PtdSer is present in multiple viable tumor cells, and its exposure is significantly increased on the surface of tumor cells or tumor cell-derived microvesicles in the tumor microenvironment, which have intrinsic immunosuppressive properties and facilitate tumor growth and metastasis.^60 61^ Our data showed that lymphoma cells expressed lower levels of Gal-9 and CEACAM1, and only moderately expressed PtdSer (online supplemental figure S3). Based on the above points, we presume that the numerous ligands of TIM-3 could easily interact with the T3/28 chimera, followed by activation of CAR-T cells. As anticipated, T3/28 CAR-T cells maintained a less-exhausted phenotype, possessed higher proliferative capacity and longer persistence on antigen challenge, and exhibited superior in vivo antitumor activity (figures 3–6). Our data indicated that enhanced cytotoxicity of T3/28 CAR-T cells was dependent on TIM-3 signaling via tumor cells at least partially. We could not exclude that ligands on CAR-T cells might bind TIM-3 in cis or in trans, which would contribute to the enhanced cytotoxicity of T3/28 CAR-T cells. Although the critical role of TIM-3 ligands in T3/28 CAR-T cells has been verified (figure 2), we did not identify the key ligand of TIM-3 that activates CD28 signaling through the T3/28 chimera, contributing to the superior cytotoxicity and persistence of T3/28 CAR-T cells.

Cumulatively, our data showed a unique role for the TIM3-CD28 chimera in maintaining higher cytotoxicity and long-term persistence of CD19-targeted CAR-T cells. Switch receptor T3/28 promotes the T_CM_ phenotype and IL-21 expression in CAR-T cells, and no additional side effects were observed. Notably, the receptor T3/28 has been preliminarily tested to improve the effect of CD138 CAR-T cells in solid tumors by substantially enhancing T-cell function and infiltration in tumors. Therefore, we propose that the switch receptor T3/28 may be widely used in engineered T cell-based immunotherapy for hematologic or solid tumors.

## Data Availability

Data are available in a public, open access repository.
