# Supplementary material for: Switch receptor T3/28 improves long-term persistence and antitumor efficacy of CAR-T cells
Source: J Immunother Cancer. 2021 Dec 1;9(12):e003176. doi: 10.1136/jitc-2021-003176 (PMC8638458; doi:10.1136/jitc-2021-003176)
Supplement: Supplementary data [file jitc-2021-003176supp001.pdf]

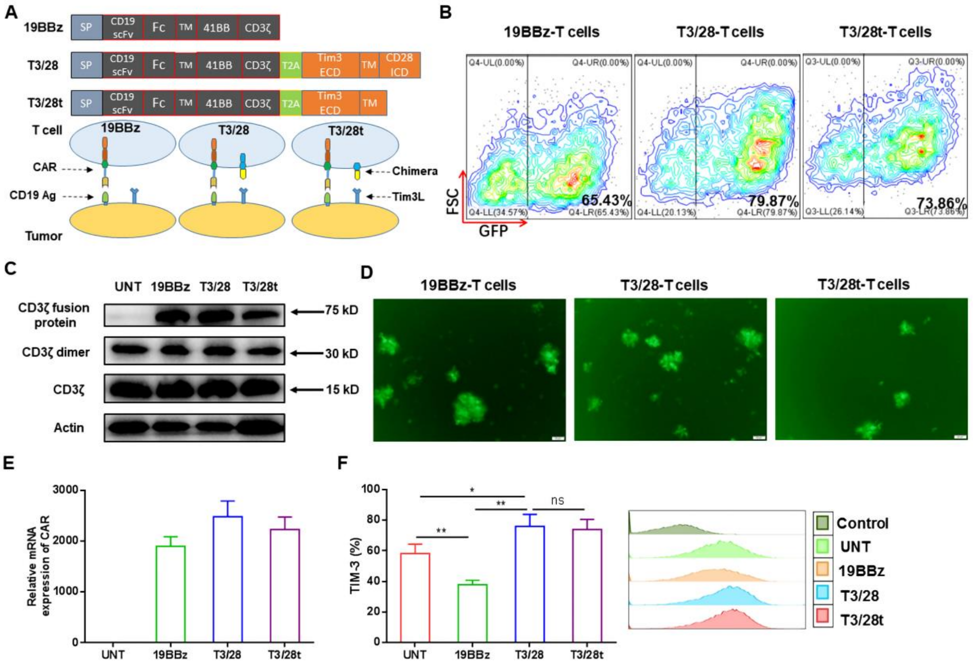

Supplementary Figure 1. Design and expression of CAR. (A) Schematic representation of the 19BBz CAR and T3/28 CAR. (B) Flow cytometry dot plots illustrating expression of 19BBz and T3/28 CAR according to GFP levels in CAR-transduced T cells. (C) Immunoblot analysis of CD3ζ fusion protein expression in 19BBz and T3/28 CAR-T cells. The molecular mass of the ζ chain was approximately 15 kDa, the molecular mass of the dimer was 30 kDa, and the molecular mass of CD3ζ fusion proteins was75 kDa. (D) Transduction efficiency of lentivirus in T cells measured by fluorescence microscope. T cells were transduced with 19BBzand T3/28 CAR. The images were taken under × 100 magnification. (E) qPCR was used to quantify expression levels of 19BBz and T3/28 CAR. (F) The expression level of TIM-3 was assayed using flow cytometry. Data presented are the mean ± SD of three separate experiments. ns means no significant difference, *p ＜0.05, **p＜0.01 compared with indicated group.

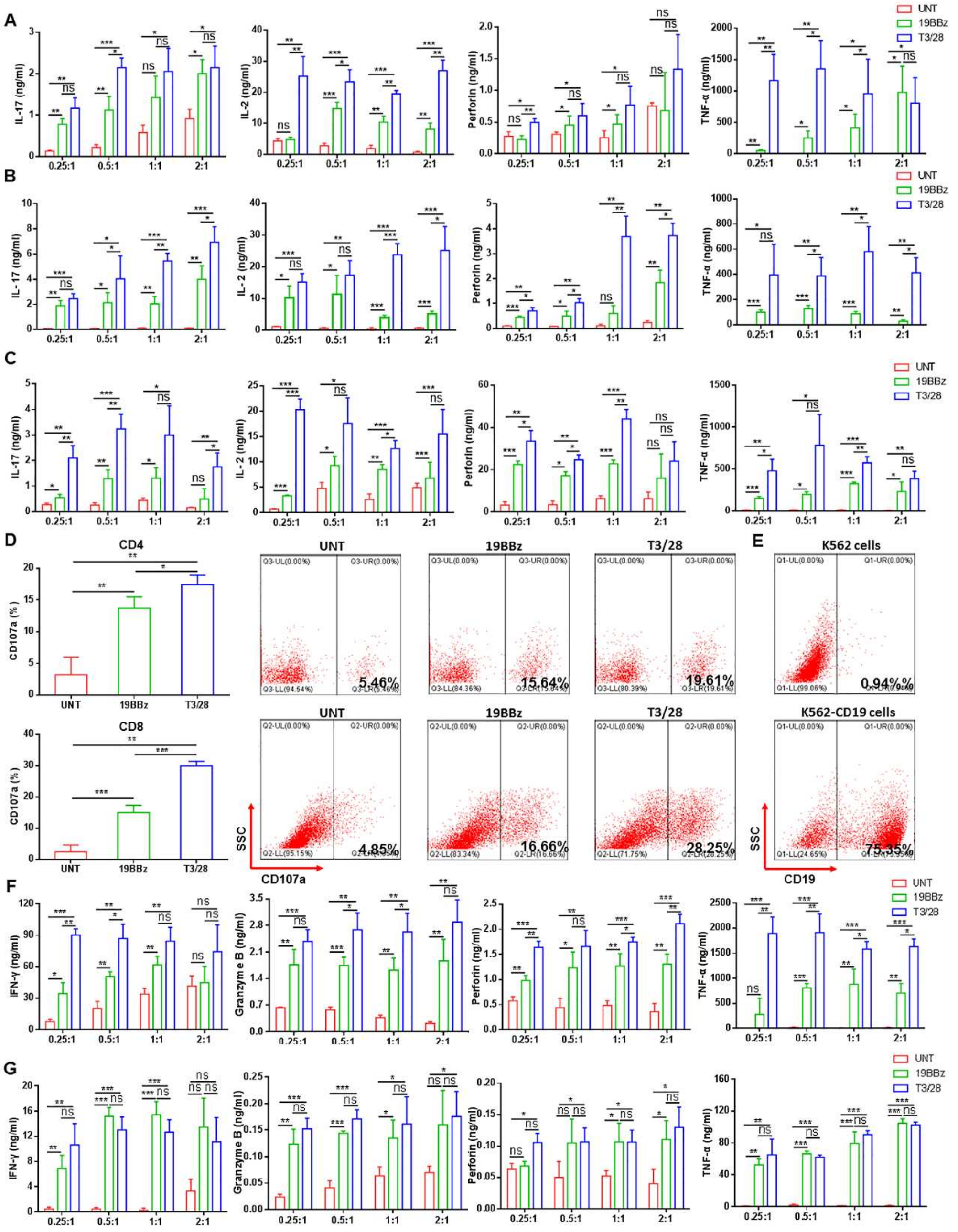

Supplementary Figure 2. The enhanced cytokine secretion of T3/28 CAR-T cells. IL-17, IL-2, perforin and TNF-α released by T cells co-cultured with Daudi (A), Raji (B) or Namalwa (C) cells were detected using microfluidic immunofluorescence technology (MIT). (D) The CD107a expression of CD4 and CD8 CAR-T cells co-cultured with Daudi cells was analyzed using flow cytometry. (E) CD19 expression on K562-CD19 cells was assayed using flow cytometry. IFN-γ granzyme B, perforin and TNF-α released by T cells co-cultured with K562-CD19 (F) and K562 (G) were measured by MIT or ELISA. Data presented are the mean ± SD of three separate experiments. ns means no significant difference, *p<0.05, **p<0.01, ***p<0.001 compared

with indicated group at the same E:T ratio.

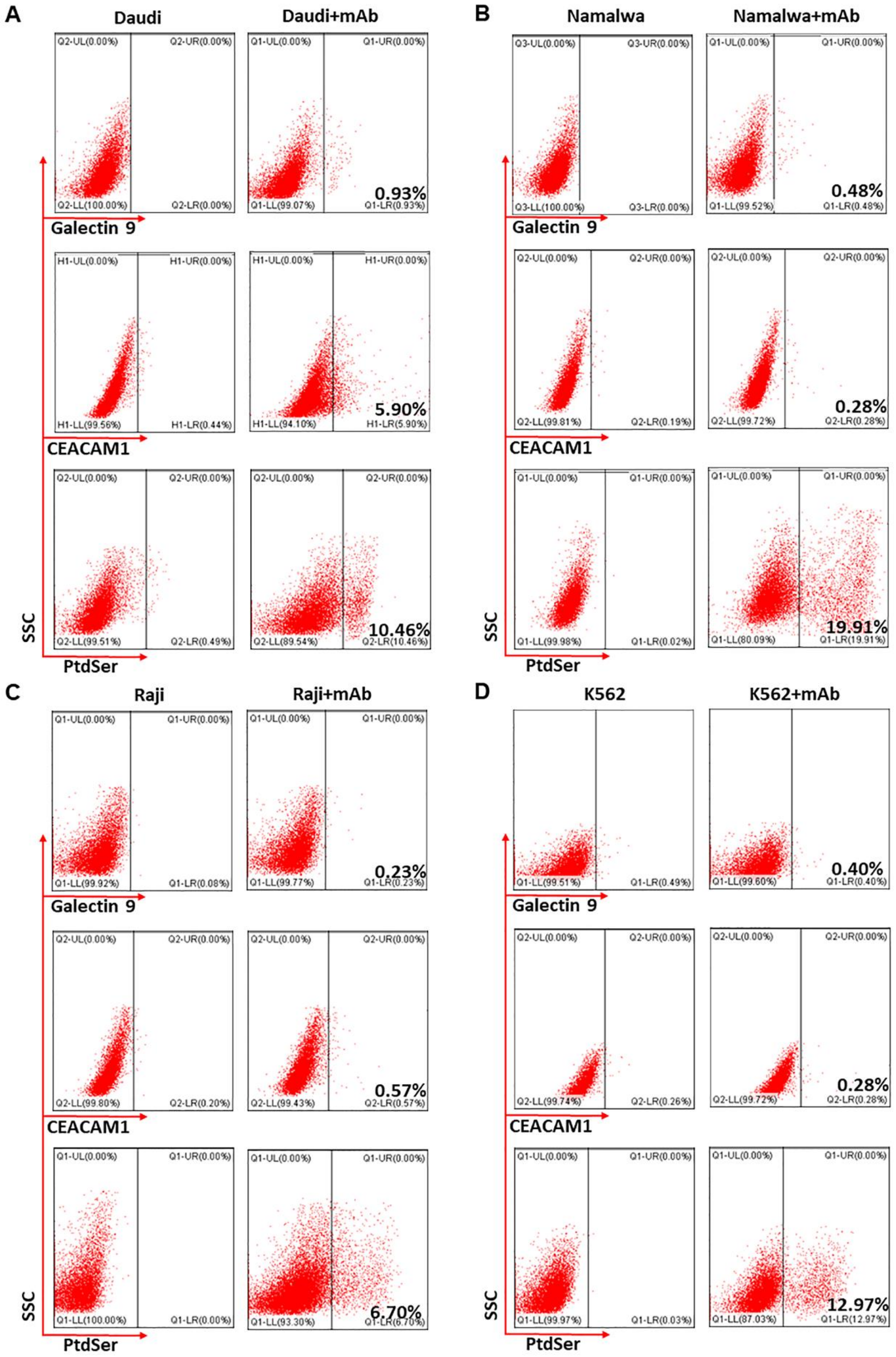

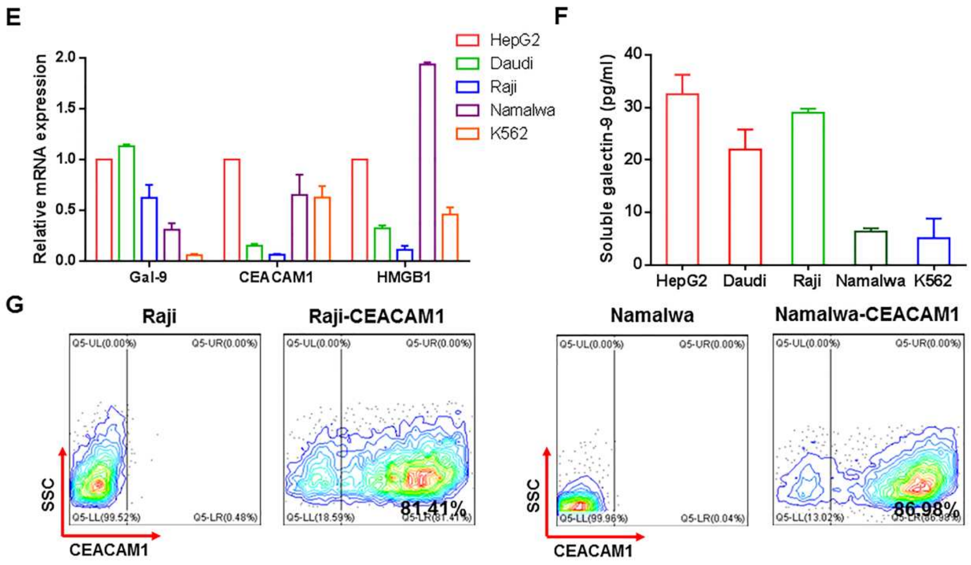

Supplementary Figure 3. The detection of TIM-3 ligands on target cells. The TIM-3 ligands Gal-9, CEACAM1 and PtdSer were analyzed on the surface of Daudi (A), Namalwa (B), Raji (C) and K562 (D) cells using flow cytometry. (E) The Gal-9, CEACAM1 and HMGB1 was analyzed using qPCR. (F) The soluble Gal-9 released by several tumor cell lines was detected. (G) CEACAM1 expression on Raji-CEACAM1 and Namalwa-CEACAM1 cells was assayed by a flow cytometer.

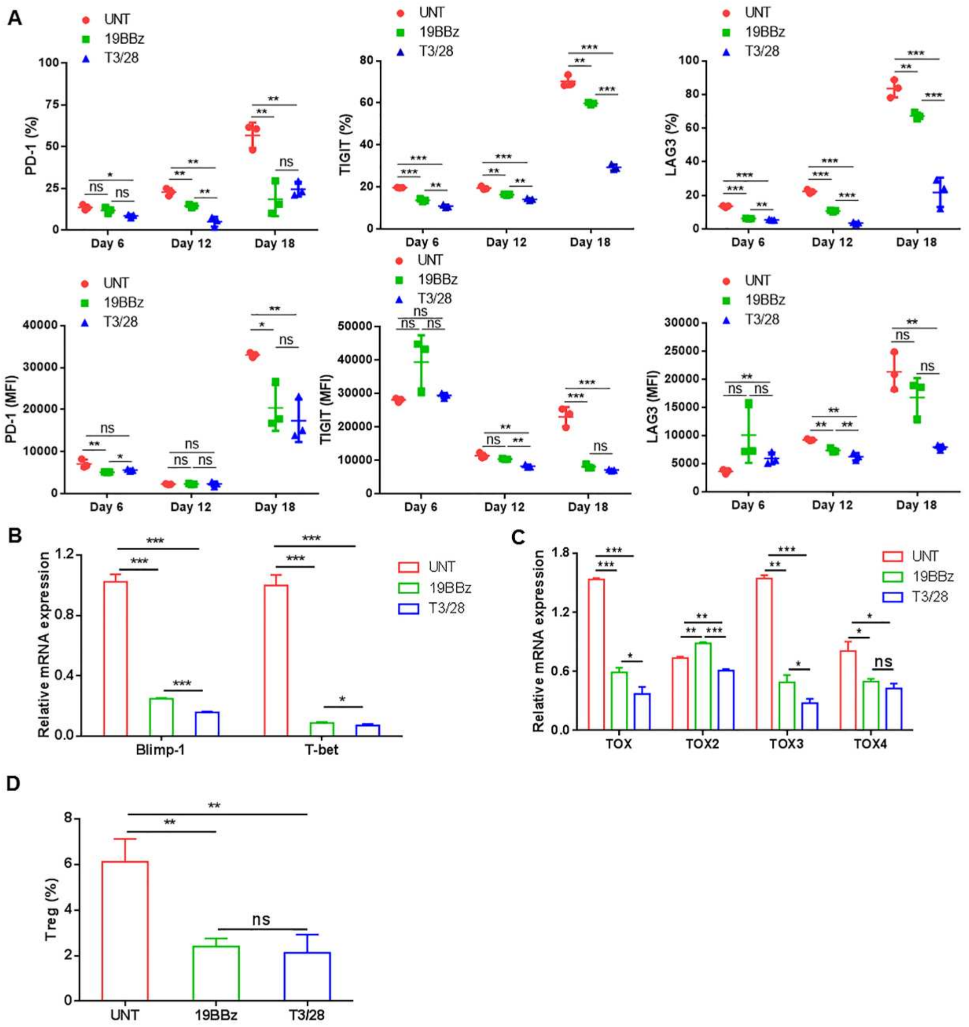

Supplementary Figure 4. Switch receptor T3/28 inhibits CAR-T cell exhaustion. (A) Expression of inhibitory receptors PD-1, TIGIT and LAG3 on CAR-T cells co-cultured with tumor cells was detected at day 6, 12 and 18 using flow cytometry. (B) qPCR analysis demonstrated a down-regulation of key exhaustion-associated transcription factors T-bet and Blimp-1 in T3/28 CAR-T cells. (C) qPCR analysis demonstrated a down-regulation of exhaustion-associated gene TOX, TOX2, TOX3 and TOX4 in T3/28 CAR-T cells. MFI: mean fluorescence intensity. (D) After *in vitro* antigen stimulation, the Treg subset of CAR-T cells was assayed at day 12 using flow cytometry. The $CD3^+CD4^+CD25^+Foxp3^+$ cells were defined as Treg. Data presented are the mean ± SD of three separate experiments. ns means no significant difference, *p＜0.05, **p＜0.01, ***p＜0.001 compared with indicated group.

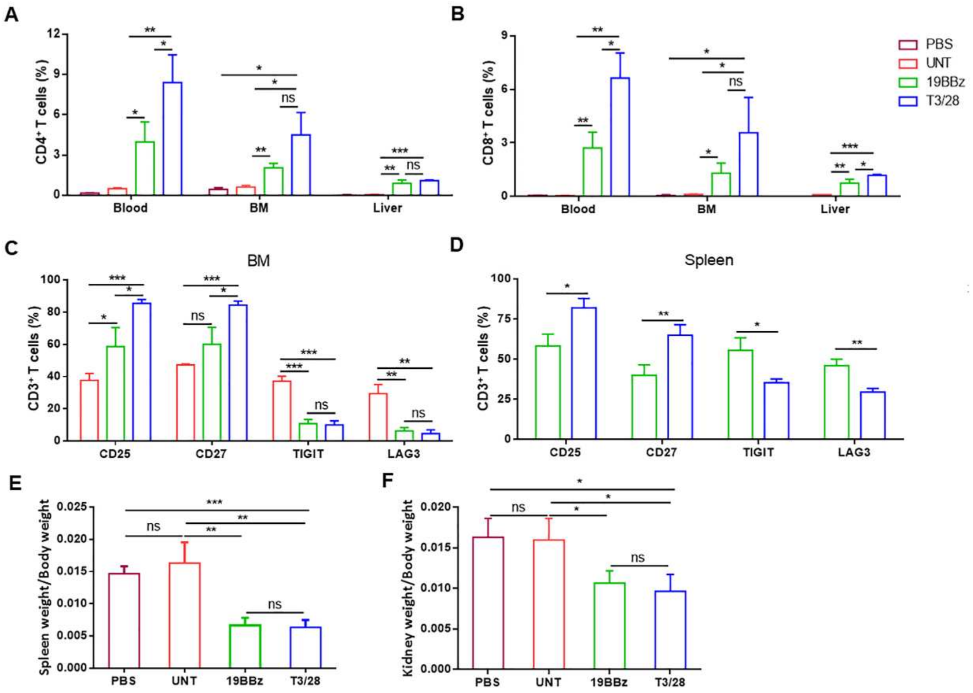

Supplementary Figure 5. Switch receptor T3/28 mediates superior anti-tumor cytotoxicity *in vivo*. The CD4⁺ CAR-T cells (A) and CD8⁺ CAR-T cells (B) from blood, BM and liver, were analyzed using flow cytometry. The expression of CD25, CD27, TIGIT and LAG3 on the surface of CD3⁺ T cells isolated from BM (C) and spleen (D) of mice was detected using flow cytometry. The liver weight: body weight ratio (E) and kidney weight: body weight ratio (F) of all groups were calculated. Data presented are the mean ± SD of three separate experiments. ns means no significant difference, *p＜0.05, **p＜0.01, ***p＜0.001 compared with indicated group. (n=4-5 mice per group).

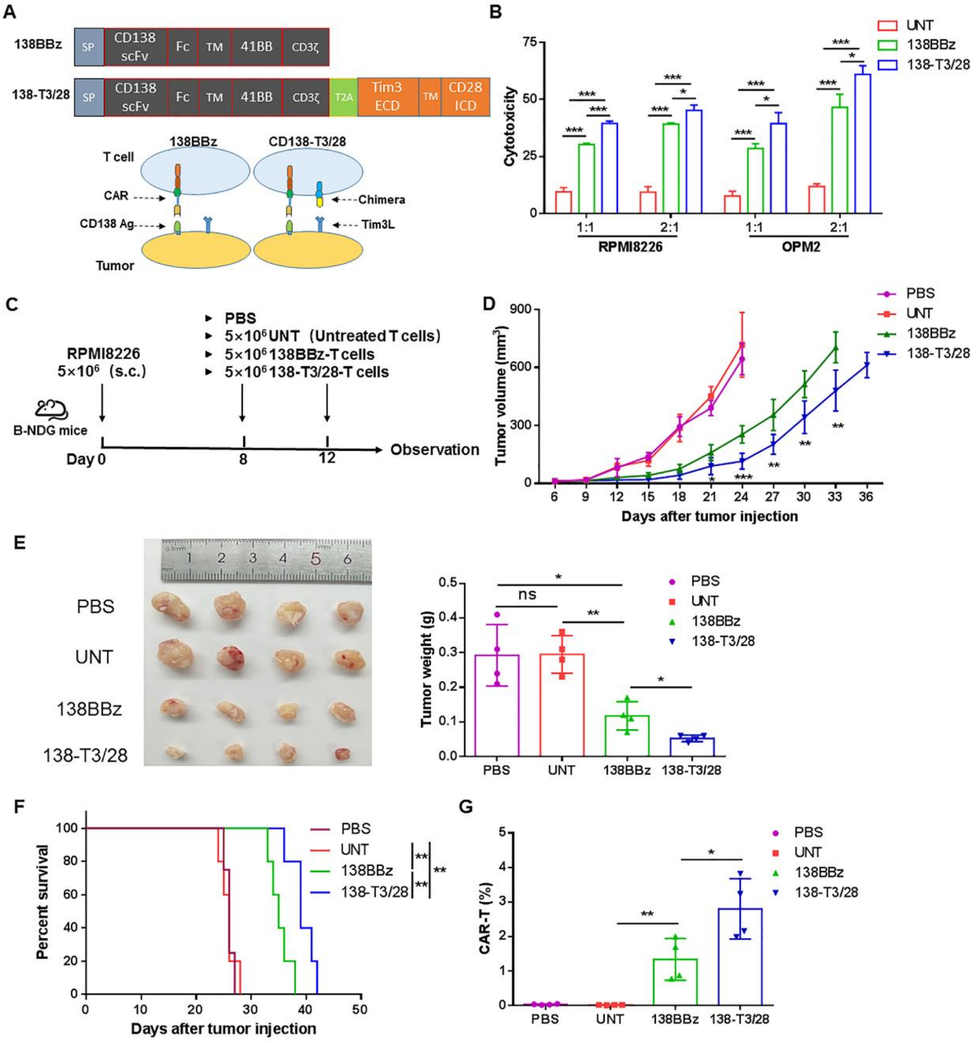

Supplementary Figure 6. CD138-T3/28 CAR-T cells mediate superior anti-CD138 positive tumor cells cytotoxicity. (A) Schematic representation of the 138BBz CAR and 138-T3/28 CAR. (B) The cytotoxicity of CAR-T or UNT cells against MM cell lines, including RPMI8226 and OPM2, was evaluated. (C) The scheme of experimental design (n=5 mice per group). (D) Tumor volume of the mice was measured. (E) Images of isolated tumors excised on day 18. (F) Survival was evaluated from the first day of tumor cell injection until death. Statistical analysis was performed using the log-rank (Mantel-Cox) text. (G) the infiltrated CAR-T cells in the tumor were analyzed. Data presented are the mean ± SD of three separate experiments. ns means no significant difference, $*p < 0.05$, $**p < 0.01$, $***p < 0.001$ compared with indicated group.

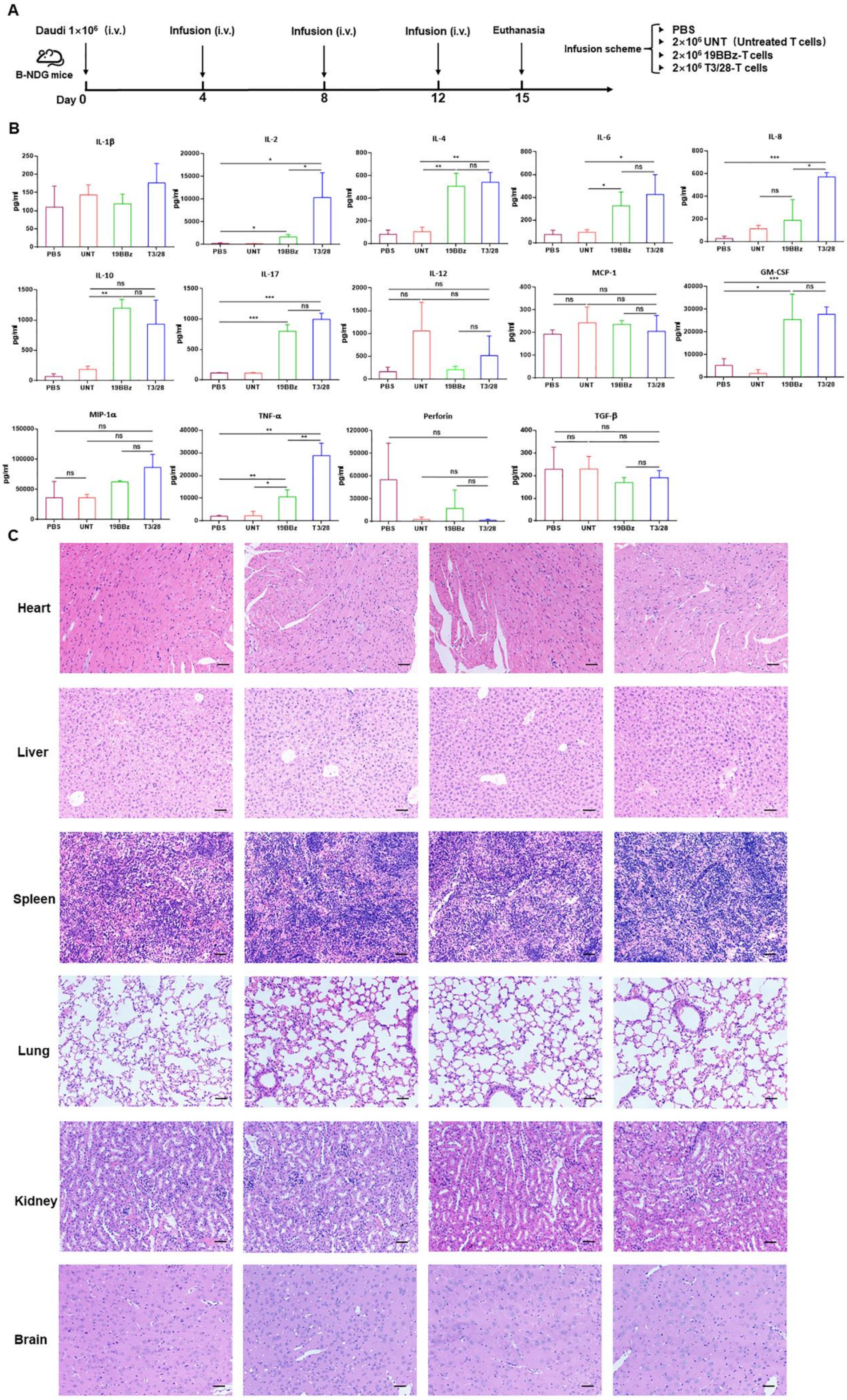
A
Daudi 1×10⁶ (i.v.)
Infusion (i.v.)
Infusion (i.v.)
Infusion (i.v.)
Euthanasia
B-NDG mice
Day 0
4
8
12
15
Infusion scheme
PBS
2×10⁶ UNT (Untreated T cells)
2×10⁶ 19BBz-T cells
2×10⁶ T3/28-T cells
B
IL-1β
IL-2
IL-4
IL-6
IL-8
IL-10
IL-17
IL-12
MCP-1
GM-CSF
MIP-1α
TNF-α
Perforin
TGF-β
pg/ml
PBS
UNT
19BBz
T3/28
C
Heart
Liver
Spleen
Lung
Kidney
Brain

Supplementary Figure 7. T3/28 CD19 CAR-T cells couldn't cause detectable CRS or evident lesions. (A) The scheme of experimental design (n=5 mice per group). (B) the CRS-related cytokines from all groups were measured using microfluidic immunofluorescence technology. (C) Tissue pathology of the indicated organs was evaluated by H&E staining at the end of the experiment. Magnification: ×400. (Scale bar 50 μm). Data presented are the mean ± SD of three separate experiments. ns means no significant difference, *p＜0.05, **p＜0.01, ***p＜0.001 compared with indicated group.

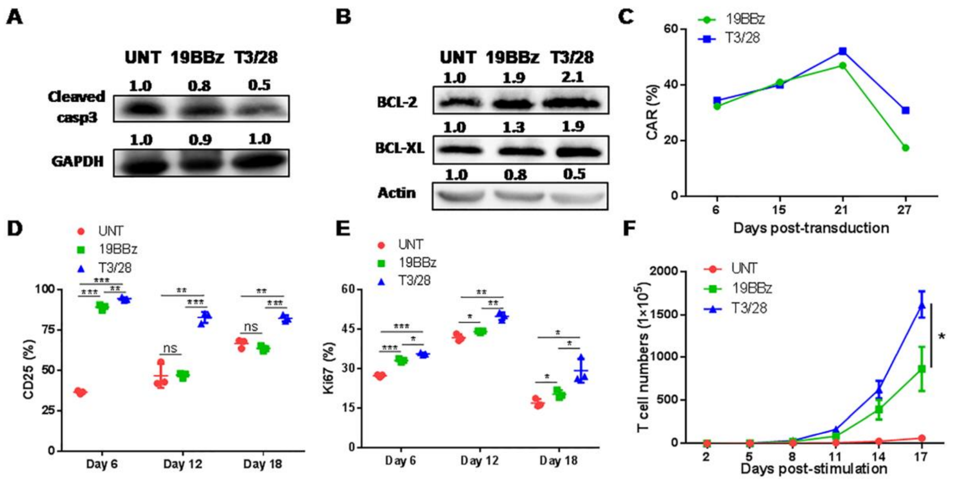

Supplementary Figure 8. Switch receptor T3/28 promotes CAR-T cell survival and proliferation. Western blot analysis was performed to detect expression of cleaved caspase-3 (A) and anti-apoptotic proteins Bcl-2 and Bcl-xL (B) in CAR-T or control cells. (C) The ratios of CAR-positive T cells were analyzed at indicated time points post transduction. Expression of proliferation marker CD25 (D) and Ki67 (E) on CAR-T cells was detected at day 6, 12 and 18 using flow cytometry. (F) The counts of UNT, 19BBz and T3/28 CAR-T cells were monitored every three days. Data presented are the mean ± SD of three separate experiments. ns means no significant difference, *p＜0.05, **p＜0.01, ***p＜0.001 compared with indicated group.

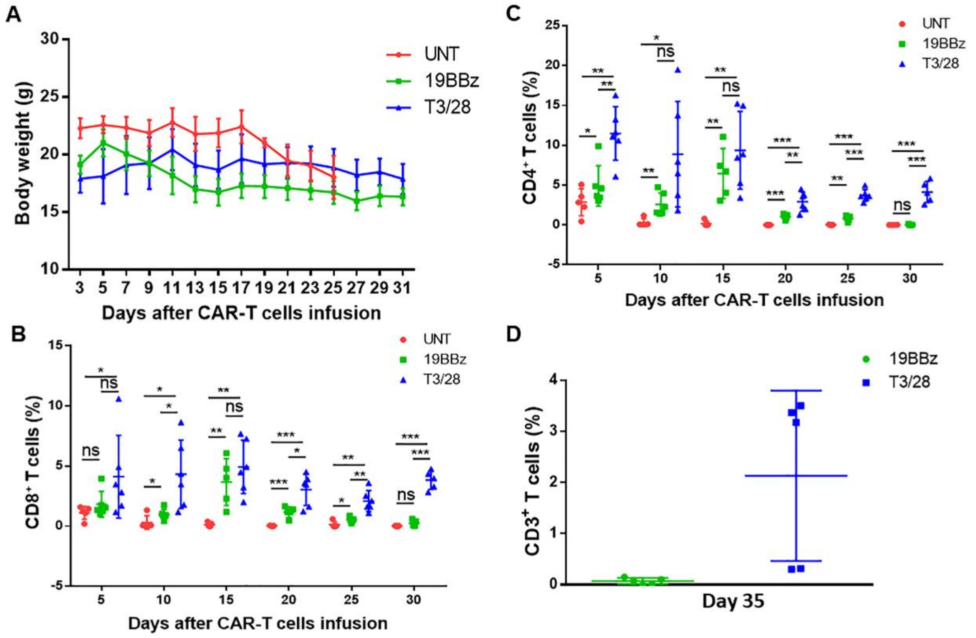

Supplementary Figure 9. T3/28 CAR-T cells possess more potent persistence ability *in vivo*. (A) Body weight change of tumor-bearing mice after CAR-T cell transfer. The CD4^{+} (B) and CD8^{+} (C) T cells from blood were monitored every 5 days. (D) the percentage of CD3^{+} CAR-T cells in peripheral blood mononuclear cells at day 35. Data presented are the mean ± SD of three separate experiments. ns means no significant difference, *p＜0.05, **p＜0.01, ***p＜0.001 compared with indicated group.

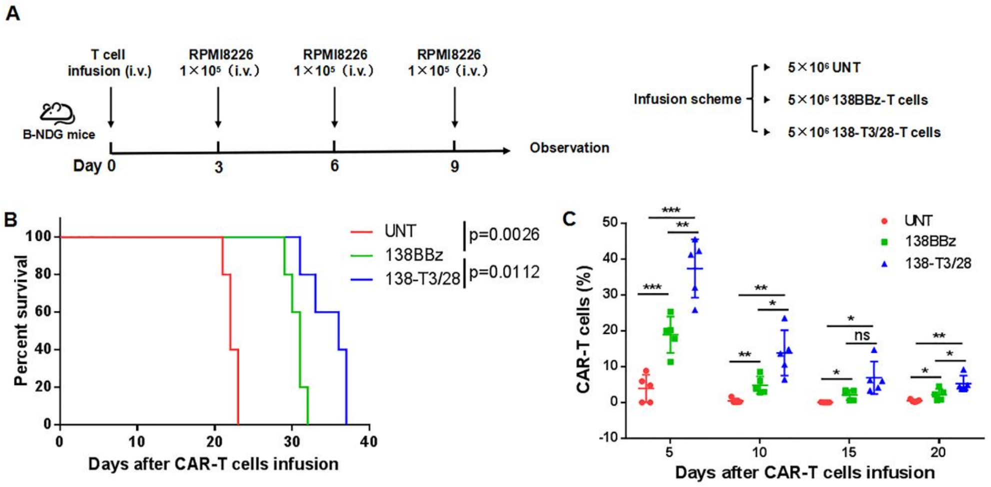

Supplementary Figure 10. CD138-T3/28 CAR-T cells possess more potent persistence ability *in vivo*. (A) The scheme of experimental design. (B) Survival was evaluated from the first day of tumor injection until death. Statistical analysis was performed using the log-rank (Mantel-Cox) text. (C) The UNT and CAR-T cells from blood were monitored every 5 days. ns means no significant difference, *p＜0.05, **p＜0.01, ***p＜0.001 compared with indicated group. (n=5 mice per group).

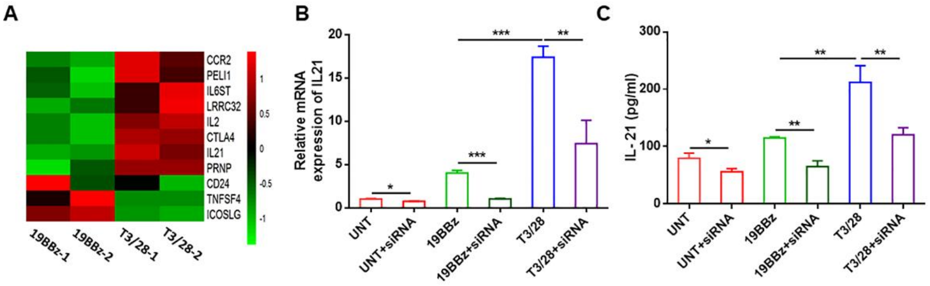

Supplementary Figure 11. T3/28 CAR-T cells produce more IL-21. (A) Heat map of RNA-sequencing analysis of 19BBz and T3/28 CAR-T cells without stimulation with antigen. IL-21expression was analyzed using qPCR (B) and ELISA (C) in different groups with or without IL-21 siRNA. Data presented are the mean ± SD of three separate experiments. *p＜0.05, **p＜0.01, ***p＜0.001 compared with indicated group.
